# Factors associated with successful vaginal birth after a cesarean section: a systematic review and meta-analysis

**DOI:** 10.1186/s12884-019-2517-y

**Published:** 2019-10-17

**Authors:** Yanxin Wu, Yachana Kataria, Zilian Wang, Wai-Kit Ming, Christina Ellervik

**Affiliations:** 1 Department of Obstetrics and Gynecology, The First Affiliated Hospital, Sun Yat-sen University, No. 58 Zhongshan Road 2, Guangzhou, 510000 People’s Republic of China; 2 Department of Laboratory Medicine, Boston Children’s Hospital and Harvard Medical School, 300 Longwood Avenue, Boston, MA 02215 USA; 3 Faculty of Health and Medical Sciences, University of Copenhagen, Copenhagen, Denmark

**Keywords:** Vaginal birth after cesarean section, Trial of labor, Delivery

## Abstract

**Background:**

Evidence for the relationship between maternal and perinatal factors and the success of vaginal birth after cesarean section (VBAC) is conflicting. We aimed to systematically analyze published data on maternal and fetal factors for successful VBAC.

**Methods:**

A comprehensive search of Medline, Embase, and the Cumulative Index to Nursing and Allied Health Literature, from each database’s inception to March 16, 2018. Observational studies, identifying women with a trial of labor after one previous low-transverse cesarean section were included. Two reviewers independently abstracted the data. Meta-analysis was performed using the random-effects model. Risk of bias was assessed by the Newcastle-Ottawa Scale.

**Results:**

We included 94 eligible observational studies (239,006 pregnant women with 163,502 VBAC). Factors were associated with successful VBAC with the following odds ratios (OR;95%CI): age (0.92;0.86–0.98), obesity (0.50;0.39–0.64), diabetes (0.50;0.42–0.60), hypertensive disorders complicating pregnancy (HDCP) (0.54;0.44–0.67), Bishop score (3.77;2.17–6.53), labor induction (0.58;0.50–0.67), macrosomia (0.56;0.50–0.64), white race (1.39;1.26–1.54), previous vaginal birth before cesarean section (3.14;2.62–3.77), previous VBAC (4.71;4.33–5.12), the indications for the previous cesarean section (cephalopelvic disproportion (0.54;0.36–0.80), dystocia or failure to progress (0.54;0.41–0.70), failed induction (0.56;0.37–0.85), and fetal malpresentation (1.66;1.38–2.01)). Adjusted ORs were similar.

**Conclusions:**

Diabetes, HDCP, Bishop score, labor induction, macrosomia, age, obesity, previous vaginal birth, and the indications for the previous CS should be considered as the factors affecting the success of VBAC.

## Background

Cesarean delivery rates have increased dramatically worldwide. In the United States, cesarean section (CS) rates increased from 5% of all deliveries in 1970 to a high of 31.9% in 2016 [1] .Although efforts were made to reduce the number of CS, it failed to achieve the 15% rate recommended by the World Health Organization (WHO) [2].

Repeat CS is the most significant factor contributing to overall increased CS rates [3]. The primary indication of repeat CS is a prior CS [3]. The trial of labor after cesarean (TOLAC) is an attempt to reduce CS rates. Several national medical associations have provided practice guidelines for vaginal birth after cesarean section (VBAC) [4, 5], but these differ across countries [6]. Generally speaking, VBAC is relatively safe when compared with repeat CS [7] .However, TOLAC rates have dropped significantly worldwide in recent years [8, 9].

For women with a prior cesarean delivery, a trial of labor will often represent her last opportunity to experience a normal birth. However, a failed VBAC increases the risk of maternal and perinatal complications more than an elective repeat CS [10] .A potential solution to the concerns related to VBAC would be a more accurate selection of patients opting for TOLAC [11]. Early communication to discuss women’s prospects for VBAC success and their attitudes towards future births might be valuable. The probability of successful vaginal birth is one of the most crucial factors in the decision-making process during the prenatal counseling of these women.

Two previous meta-analyses were published in 1990 (Rosen et al.) and in 2010 (Eden et al.). Rosen et al. focused on the indicators in the previous cesarean for VBAC success [12]. Eden et al. focused on studies about predictors of VBAC, which were conducted in developed countries [11]. They found that cephalopelvic disproportion (CPD) in the previous cesarean, previous breech, previous vaginal delivery, more than one previous cesarean, Hispanic ethnicity, advanced age, birth weight heavier than 4 kg, and use of either augmentation or induction affected the likelihood of VBAC. However, no previous meta-analysis has focused on the influences of obesity, diabetes, hypertensive disorders complicating pregnancy (HDCP), gestational weeks, and interdelivery interval on the chance of VBAC, which were conflicting.

Therefore, we aimed to perform a systematic review and meta-analysis of all published reports until 2018 of vaginal birth after one previous cesarean and maternal or fetal factors from the historical and current pregnancies, and to quantify the magnitude of each factor and the quality of the supporting data.

## Methods

The study was conducted in accordance with the Meta-analysis of Observational Studies in Epidemiology (MOOSE) recommendations (Additional file 22: Text S1). We have reported our findings following the Preferred Reporting Items for Systematic Reviews and Meta-Analyses (PRISMA) reporting guidelines (Additional file 23: Text S2). We submitted the protocol to Prospero before initiation of the analyses (ID: CRD42018087395).

### Literature search

A search for the following sources was performed from database inception until March 16, 2018: Medline, Embase, and Cumulative Index to Nursing and Allied Health Literature. We used Medical Subject Headings, keywords and word variants for the trial of labor, vaginal birth and cesarean in the search strategy, with the help of an experienced librarian. The search strategy is outlined in the Additional file 24: Appendix 1. Bibliographies of selected review articles were reviewed for additional relevant studies. Only studies about human data published in or translated into English were included. The Countway Library of Medicine at Harvard Medical School assisted with crafting and implementing the literature search,

### Exposure

Exposure was defined as maternal or fetal factors for VBAC. Factors related to previous pregnancies included: previous vaginal birth (VB) before CS, previous VBAC, and indications for the previous CS. Factors from the current pregnancy included: age (year), body mass index (BMI, kg/m^2^), obesity (BMI ≥ 30 kg/m^2^), smoke, race (White, Asian, Black, Latina), diabetes (pre-existing, gestational diabetes mellitus), hypertensive disorders complicating pregnancy (HDCP), interdelivery interval (between the last two pregnancies), gestational weeks, Bishop score at admission before delivery, labor induction, epidural anesthesia during labor, and macrosomia (birth weight ≥ 4 kg).

### Outcome

TOLAC was defined as an attempt at vaginal delivery after a previous cesarean section. A successful VBAC is defined as spontaneous or instrumental (assisted by vacuum or forceps) delivery to a woman undergoing TOLAC. A failed VBAC is defined as failure to achieve a VBAC and the delivery ending by emergency cesarean section. In the study, all of the pregnant women had experienced TOLAC, and were grouped as successful VBAC or failed VBAC.

### Inclusion criteria

The target population of the study included women of child-bearing ages, with a single gestation and, one previous cesarean delivery, and that were candidates for attempted vaginal birth.

The following criteria were required for eligibility: 1) mean and SD of the continuous factors and N in women with successful VBAC and failed VBAC, or 2) unadjusted and/ or adjusted odds ratio (OR) and 95% confidence interval (CI) for the binary factors in women with successful VBAC and failed VBAC, or 3) raw N for the 2*2 tables to calculate the OR and 95% CI for the binary factors in women with successful VBAC and failed VBAC.

### Exclusion criteria

We excluded women with more than one previous cesarean delivery; known previous classical uterine incision or T-incision, prior uterine rupture, or extensive transfundal uterine surgery, multiple gestations, and those in whom vaginal delivery is otherwise contraindicated (e.g, those with placenta previa) [13].

### Data extraction

Two authors (YW and YK) examined studies on the basis of inclusion and exclusion. Studies were initially reviewed on titles and abstracts, and those deemed relevant were reviewed in full text. Disparities in selection were resolved through discussion and ultimately by the third reviewer (CE). In cases of study duplication, the more recent studies were selected for inclusion. Data were extracted by two authors (YW and YK) to verify the accuracy.

### Assessment of risk of bias

The quality of each study was evaluated and scored independently by two authors (YW and CE) using the nine-star Newcastle-Ottawa Scale (NOS). Studies were evaluated based on selection, comparability, exposure, and outcome, and scored by a maximum of nine points. Scores above five indicate moderate to high study quality. The NOS for cohort and case-control studies was retrieved from: http://www.ohri.ca/programs/clinical_epidemiology/nosgen.pdf.

### Data synthesis

From each study, we extracted or estimated the odds ratio (OR) for each factor and outcome of interest, with the 95% confidence intervals (CI). We also extracted mean and SD for continuous variables of each factor. We used the statistical program Stata 14.0 and the commands “metan” to calculate random effects summary estimates. For continuous variables we used standardized mean differences (SMD) with 95% CI. For binary variables, we used ORs and 95% CIs. Statistical heterogeneity was assessed graphically with forest plots and statistically using Cochran’s Q-statistic and the I^2^ value. Publication bias was measured by Egger’s test and by visually assessing funnel plots. YW and CE were responsible for the data synthesis.

## Results

Ninety-four studies were ultimately included in our analysis [9, 14–106]. The study selection process is shown in Additional file 1: Figure S1. A total of 94 studies were included in our analysis. Twenty-eight were prospective cohort studies, and 66 were retrospective observational studies. No randomized trials were identified. The number of women in the included studies ranged from 28 to 75,086 (Additional file 18: Table S1). Details of the selected studies and the data extracted from each study were shown in Additional file 19: Table S2. The Newcastle–Ottawa Scale revealed a score of 7–8 in case-control studies and 6–9 in cohort studies (Additional file 20: Table S3). In total, 239,006 women who attempted a TOLAC were included; the successful rate of VBAC was 68.4%. Compare to the other continents (Additional file 2: Figure S2), women in African region achieved the lowest successful rate (54.1%) of VBAC (*P* < 0.05).

**Fig. 1:**
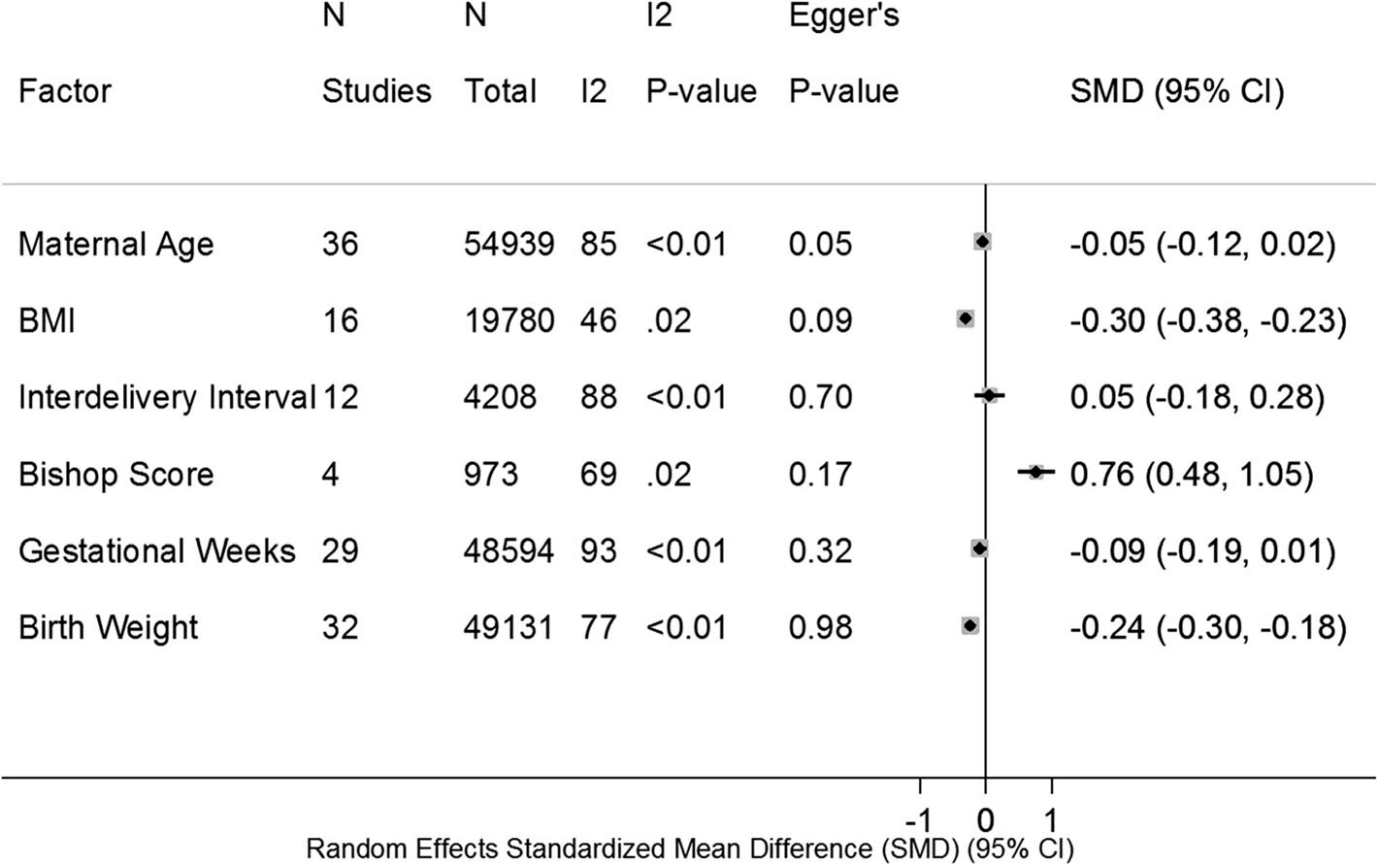
Maternal and Fetal Factors and VBAC – SMD. *SMD, Standardized Mean Difference

**Fig. 2:**
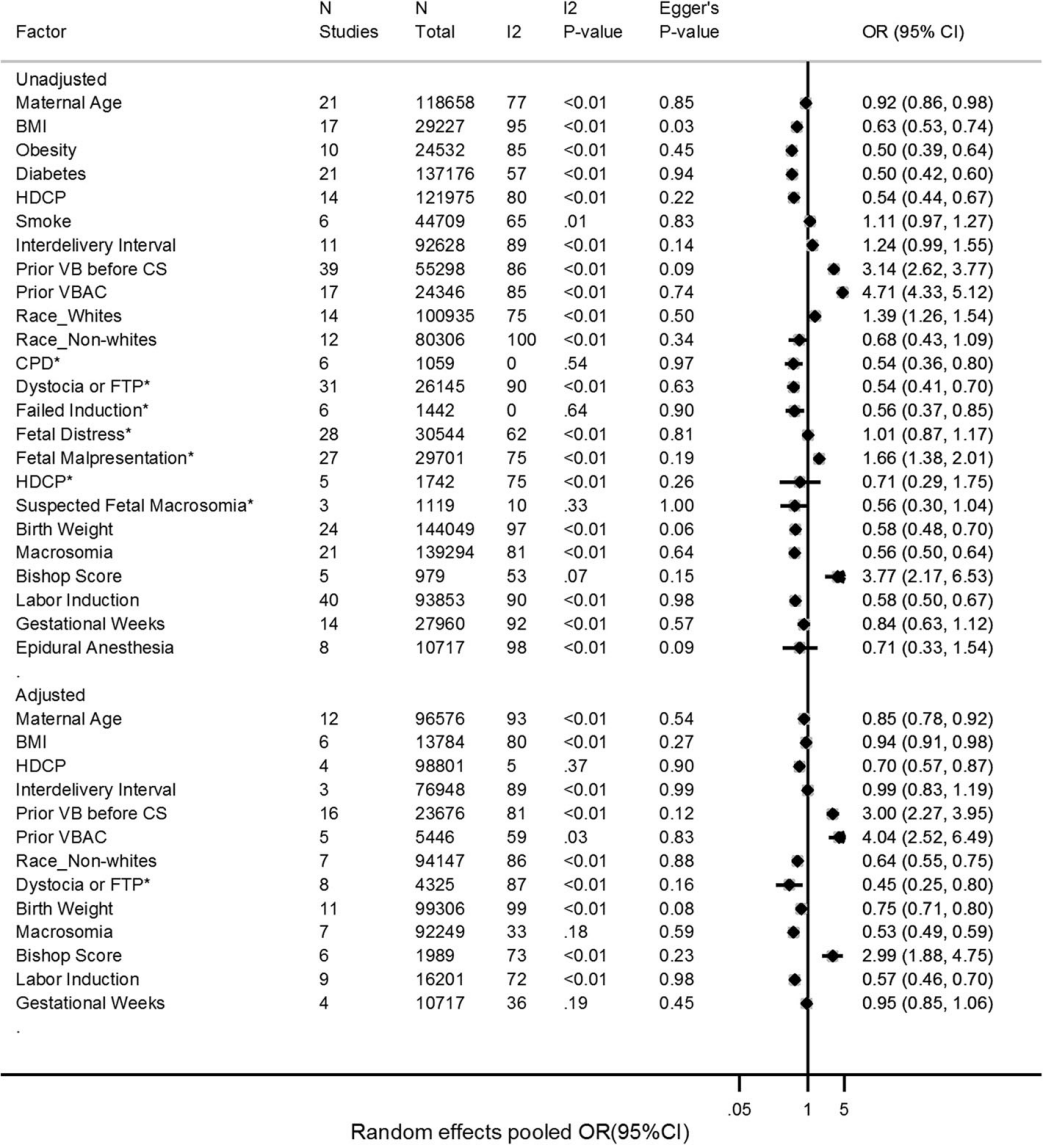
Factors associated with VBAC – OR. *OR, odds ratio

Continuous variable differences of maternal and fetal factors between successful VABC and failed VBAC are shown in Fig. 1. The associations of maternal and fetal factors with the success of VBAC are shown in Fig. 2.

Significant SMD of factors for VBAC were the following: BMI (SMD,-0.30; 95% CI: − 0.38, − 0.23) (Additional file 3: Figure S3), Bishop score (SMD, 0.76; 95% CI: 0.48, 1.05) (Additional file 5: Figure S5A), birth weight (SMD,-0.24; 95% CI: − 0.30, − 0.18) (Additional file 6: Figure S6A).

Factors associated with statistically significant increased likelihood of VBAC were the following: previous VB before CS (OR, 3.14; 95% CI, 2.62–3.77; adjusted OR, 3.00; 95% CI, 2.27–3.95) (Additional file 7: Figure S7A and S7B), previous VBAC (OR, 4.71; 95% CI, 4.33–5.12; adjusted OR, 4.04; 95% CI, 2.52–6.49) (Additional file 8: Figure S8A and S8B), Bishop score at admission before delivery (OR, 3.77; 95% CI, 2.17–6.53; adjusted OR, 2.99; 95% CI, 1.88–4.75) (Additional file 5: Figure S5B and S5C), fetal malpresentation as the indication for previous CS (OR, 1.66; 95% CI, 1.38–2.01) (Additional file 9: Figure S9D), and White race (OR, 1.39; 95% CI, 1.26–1.54) (Additional file 10: Figure S10A).

**Fig. 3:**
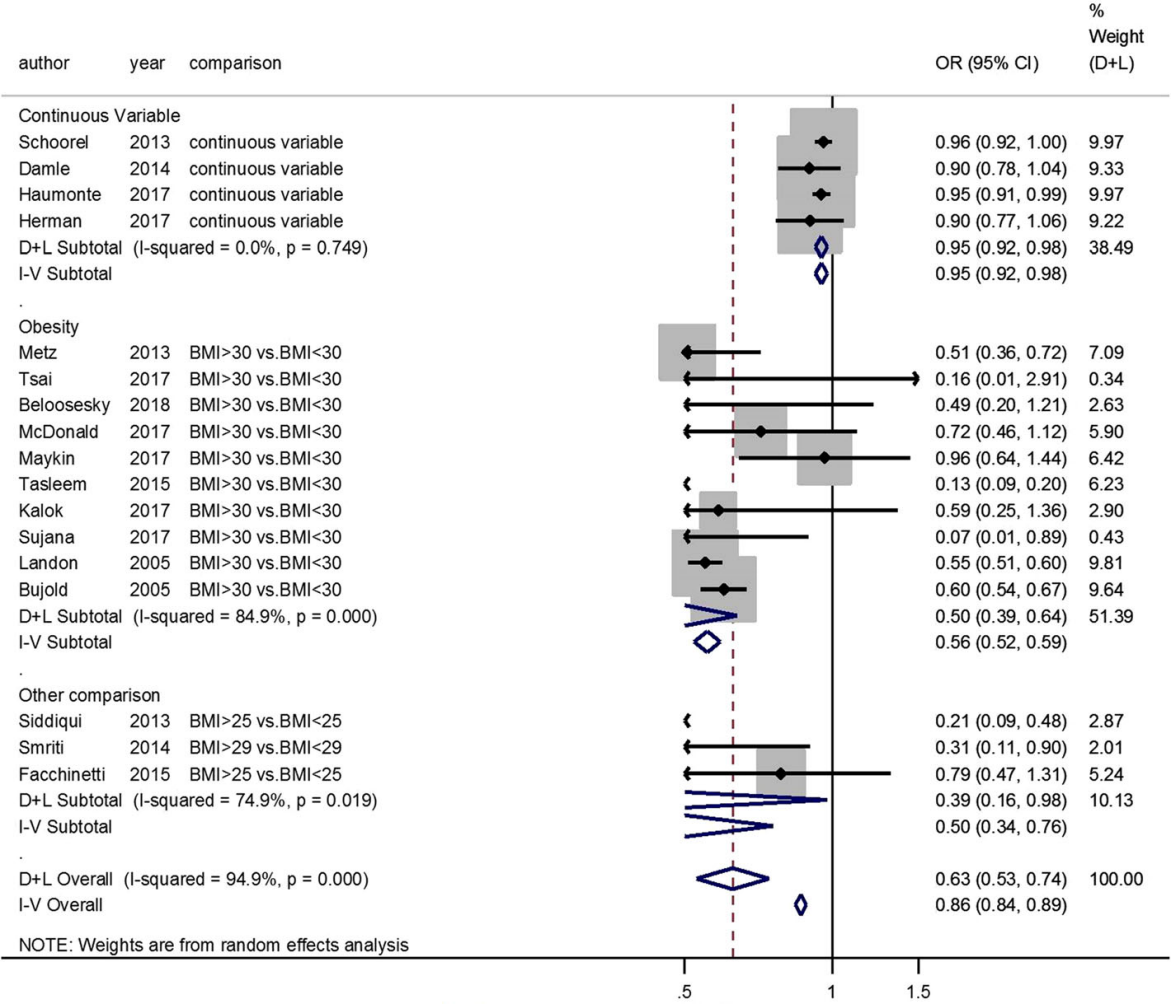
Body mass index (BMI) and VBAC

**Fig. 4:**
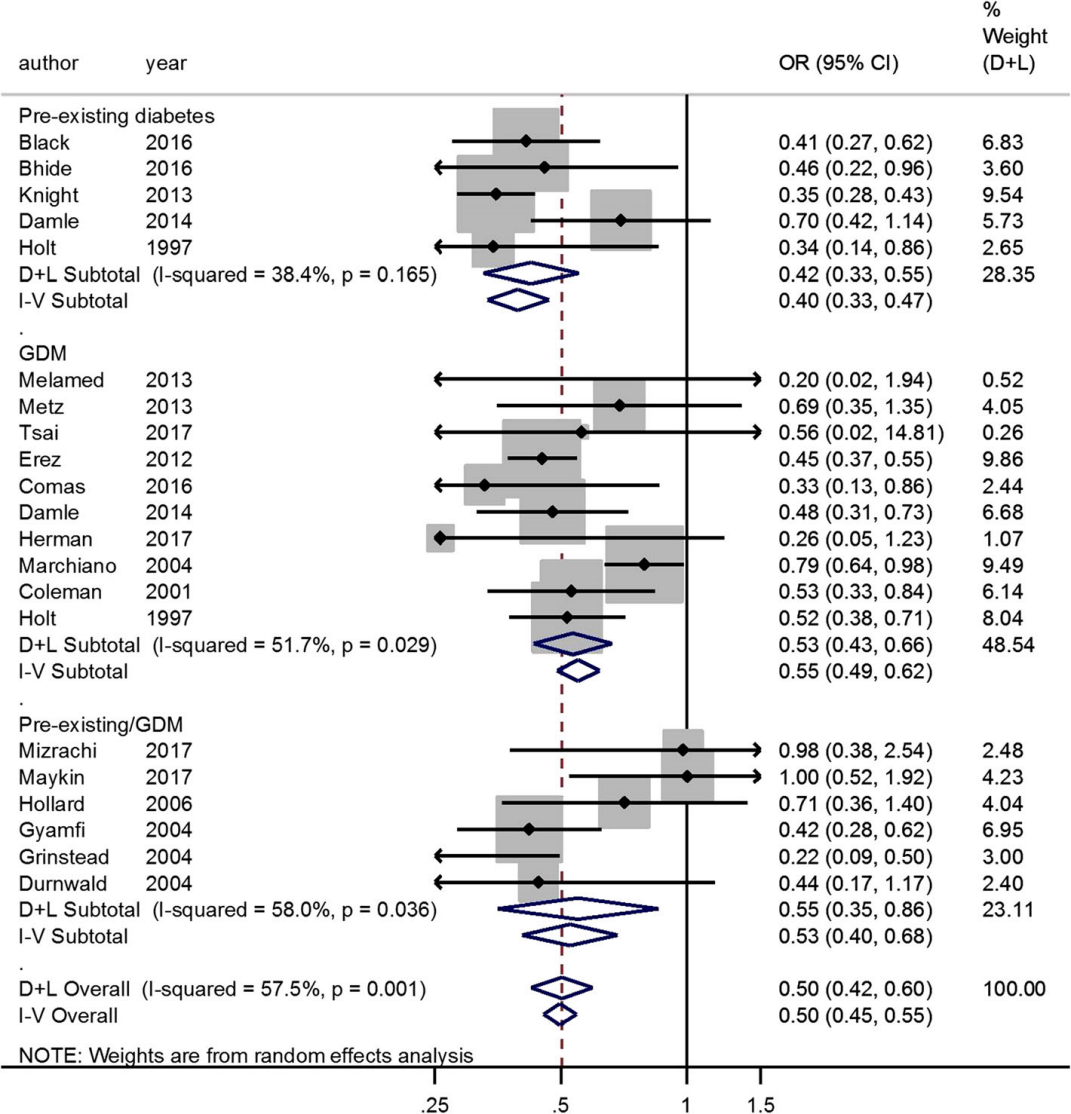
Diabetes and VBAC. *GDM, gestational diabetes mellitus

**Fig. 5:**
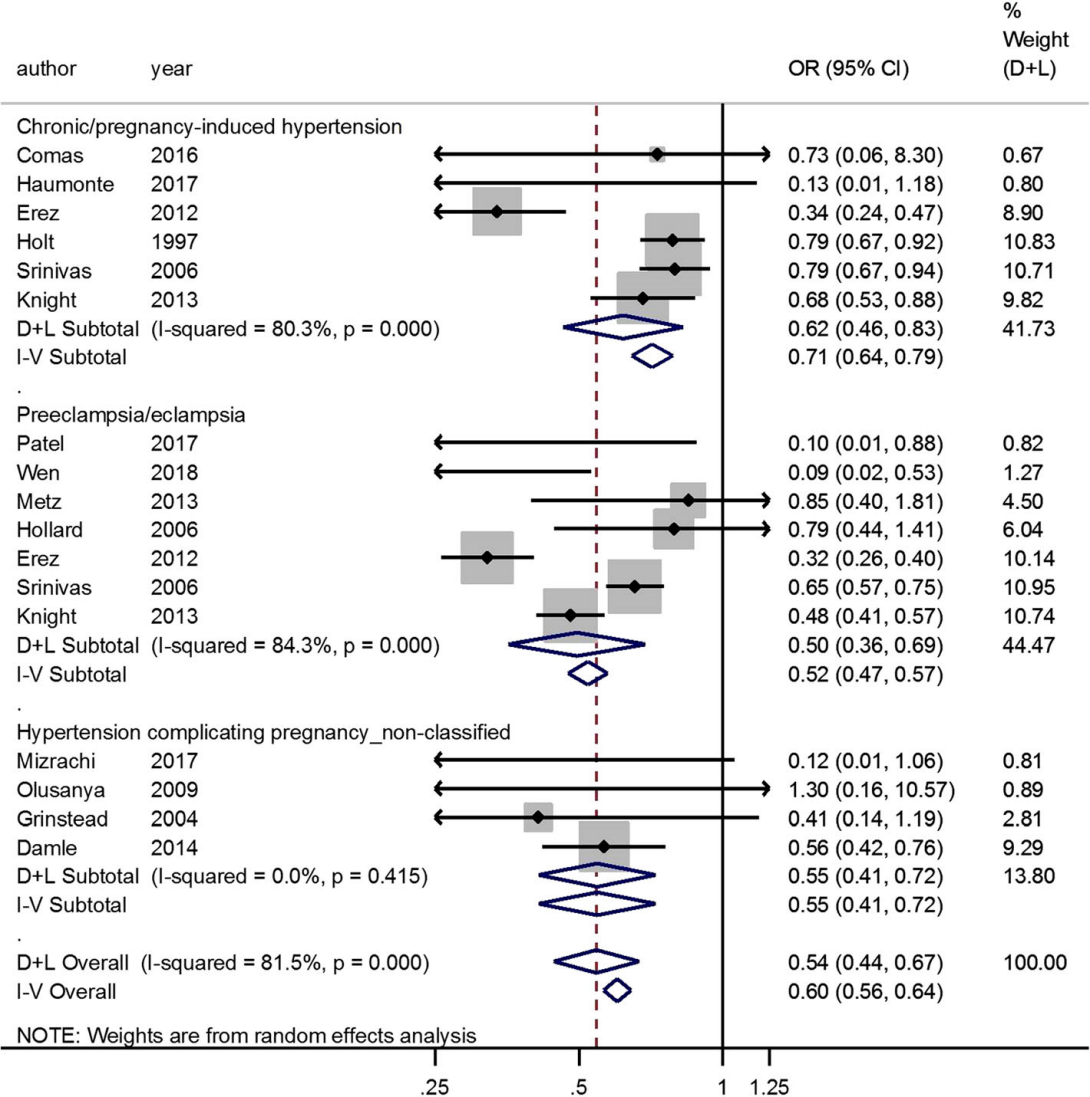
Hypertensive disease complicating pregnancy and VBAC

Risk factors associated with statistically significant decreased likelihood of VBAC were the following: Age (OR, 0.92; 95% CI, 0.86–0.98; adjusted OR, 0.85; 95% CI, 0.78–0.92) (Additional file 11: Figure S11B and S11C), BMI (OR, 0.63; 95% CI, 0.53–0.74; adjusted OR, 0.94; 95% CI, 0.91–0.98) (Fig. 3 and Additional file 4: Figure S4), diabetes (OR, 0.50; 95% CI, 0.42–0.60) (Fig. 4), HDCP (OR, 0.54; 95% CI, 0.44–0.67; adjusted OR, 0.70; 95% CI, 0.57–0.87) (Fig. 5 and Additional file 12: Figure S12), macrosomia (OR, 0.56; 95% CI, 0.50–0.64; adjusted OR, 0.53; 95% CI, 0.49–0.59) (Additional file 6: Figure S6B and S6C), labor induction (OR, 0.58; 95% CI, 0.50–0.67; adjusted OR, 0.57; 95% CI, 0.46–0.70) (Additional file 13: Figure S13A and S13B), Black race compared to the White (OR, 0.54; 95% CI, 0.19–1.54; adjusted OR, 0.51; 95% CI, 0.44–0.60) (Additional file 10: Figure S10B and S10C), Asian race compared to the White (OR, 0.67; 95% CI, 0.50–0.90; adjusted OR, 0.72; 95% CI, 0.58–0.90) (Additional file 10: Figure S10B and S10C), Latina race compared to the White (OR, 0.71; 95% CI, 0.56–0.89; adjusted OR, 0.70; 95% CI, 0.50–1.00) (Additional file 10: Figure S10B and S10C), and the indications for previous CS such as cephalopelvic disproportion (CPD) (OR, 0.54; 95% CI, 0.36–0.80) (Additional file 9: Figure S9A), dystocia or failure to progress (OR, 0.54; 95% CI, 0.41–0.70; adjusted OR, 0.33; 95% CI, 0.27–0.40) (Additional file 9: Figure S9B and S9E), and failed induction (OR, 0.56; 95% CI, 0.37–0.85) (Additional file 9: Figure S9A).

Factors not associated with likelihood of VBAC were the following: smoke (OR, 1.11; 95% CI, 0.97–1.27) (Additional file 14: Figure S14), interdelivery interval (OR, 1.24; 95% CI, 0.99–1.55; adjusted OR, 0.99; 95% CI, 0.83–1.19) (Additional file 15: Figure S15B and S15C), gestational weeks (OR, 0.84; 95% CI, 0.63–1.12; adjusted OR, 0.95; 95% CI, 0.85–1.06) (Additional file 16: Figure S16B and S16C), epidural anesthesia (OR, 0.71; 95% CI, 0.33–1.54) (Additional file 17: Figure S17), and the indications for previous CS (fetal distress (OR, 1.01; 95% CI, 0.87–1.17) (Additional file 9: Figure S9C), HDCP (OR, 0.71; 95% CI, 0.29–1.75) (Additional file 9: Figure S9A), and suspected fetal macrosomia (OR, 0.56; 95% CI, 0.30--1.04) (Additional file 9: Figure S9A).

We also performed the analysis by subgroup for age, BMI, diabetes, and HDCP. Advanced age (age ≥ 35 years-old) is associated with VBAC failure (OR, 0.97; 95% CI, 0.85–1.11; adjusted OR, 0.75; 95% CI, 0.65–0.86) (Additional file 11: Figure S11B and S11C). Pre-pregnancy BMI, BMI at first prenatal visit or BMI at admission before delivery of women with successful VBAC were lower than those of women with failed VBAC (Additional file 3: Figure S3). Obesity (BMI ≥ 30 kg/m^2^) was a risk factor for failed VBAC (OR, 0.50; 95% CI, 0.39–0.64) (Fig. 3). Both pre-existing diabetes (OR, 0.42; 95% CI, 0.33–0.55) and gestational diabetes mellitus (GDM) (OR, 0.53; 95% CI, 0.43–0.66) were identified as risk factors for failed VBAC (Fig. 4). The trends were similar in chronic/pregnancy-induced hypertension (OR, 0.62; 95% CI, 0.46–0.83) and preeclampsia/eclampsia (OR, 0.50; 95% CI, 0.36–0.69) (Fig. 4).

### Heterogeneity and publication Bias and sensitivity analysis

Almost all point estimates were in the same direction, and I^2^ heterogeneity < 50% was detected in the following meta-analysis: OR and adjusted OR of advanced age, SMD of BMI (pre-pregnancy or at admission before delivery), OR of pre-existing diabetes, adjusted OR of HDCP, adjusted OR of macrosomia, OR and adjusted OR of previous VBAC, adjusted OR of Black race, adjusted OR of Asian race, adjusted OR of Latina race, OR of Bishop score, adjusted OR of gestational weeks, and OR of indications for previous CS (such as CPD, failed induction, and suspected fetal macrosomia). The rest of the meta-analyses had either point estimates in both directions or I^2^ heterogeneity > 50%. If I^2^ heterogeneity > 50% and *P*-value < 0.05, the meta-analysis was stratified by study size, location and study design. After stratifying by subgroup, most I^2^ heterogeneities became lower (Additional file 21: Table S4). Except for the SMD of age and OR of BMI, there was no evidence of publication bias in the meta-analysis (Figs. 1 and 2).

## Discussion

### Main findings

Based on the meta-analyses, the following factors were associated with a successful VBAC: previous VB before CS, previous VBAC, White race, higher bishop score, and fetal malpresentation as the indication for previous CS. The following factors were associated with an unsuccessful VBAC: advanced age, obesity, diabetes, HDCP, non-white race, macrosomia, labor induction, and CPD, dystocia or FTP, failed induction as the indications for previous CS. The following factors are not statistically associated with VBAC success: smoke, interdelivery interval, gestational weeks, epidural anesthesia during labor, and the indications for previous CS (fetal distress, HDCP, and suspected fetal macrosomia).

Understanding the influences of factors on VBAC could provide sufficient evidence to assess chances for achieving a successful vaginal delivery among women with prior CS. It also could help the clinician provide evidence-based counseling about VBAC, which has important implications on avoiding repeated CS. The evidence quality was considered moderate on the Newcastle-Ottawa Scale.

### Strengths and limitations

This meta-analysis is the most recent that comprehensively, critically, and quantitatively assesses the association between maternal or fetal factors and the chances for achieving a successful VBAC, including factors both from the historical and current pregnancies. It is also the first large study to specifically evaluate the association between obesity, diabetes, HDCP, gestational weeks, interdelivery interval and VBAC success. However, this study has limitations. First, there might be pertinent studies that were not identified owing to inherent limitations in database literature searches. Second, the quality of evidence is limited by data largely derived from retrospective observational analyses, with heterogeneous data reporting and study design. Third, after subgroup analyzed by study size, location, and study design, I^2^ heterogeneity was still high in some meta-analysis, which might result from the change of the guidelines on VBAC or guidelines varying in different countries. Forth, there was publication bias in the meta-analyses of the SMD of age and the OR of BMI, which might be because positive results are more likely to be published compared to null studies and almost these studies showed the similar trends of the factors related to VBAC. Five, some studies did not present adjusted OR; however, the pooled unadjusted OR was similar to the adjusted OR.

### Interpretation

#### Historic maternal and fetal factors

Having experienced a vaginal birth (VB), either before CS or after CS, tripled the success rates of VBAC. Moreover, the previous VBAC was a stronger predictor of success than previous VB before CS. These findings can further confirm that the history of past vaginal deliveries is of the utmost importance. Previous VB also related to the lower rate of uterine rupture [18].

Indications for previous CS have important implications for the chance for VBAC. CPD, dystocia or failure to progress, failed induction, and suspected fetal macrosomia were risk factors for failed VBAC. Although the above conditions might not be present in the next pregnancy, the indications for previous CS could help us to identify the VBAC candidates.

#### Current maternal and fetal factors

Increased age decreases the likelihood of VBAC. Women with advanced age were more likely to fail to VBAC, which was also supported by Eden et al. [11] Age ≥ 40 years-old was also a risk for uterine rupture when women undertook TOLAC [107]. So, younger women, especially those < 35-years-old, are more likely to have a successful and safe VBAC.

Maternal obesity carries the risk for many obstetric complications including macrosomia and increased risk of CS [108]. Both obesity and macrosomia have negative impacts on VBAC success. When comparing cases where obesity occurred at pre-pregnancy or at admission before delivery, the trends are similar. Faucett et al. found that women with obesity were more likely to undergo emergency cesarean for an arrest disorder before achieving active labor despite having more clinical interventions to achieve a vaginal birth [109]. A better understanding of the mechanisms by which maternal obesity affects the progression of labor, might help to increase the rates of successful VBAC among this population. Maternal obesity was also associated with a high risk of uterine dehiscence or rupture at term gestation among women with previous CS [110]. Therefore, appropriate weight and weight gain during pregnancy are vital for maternal health.

Gestational and pregestational diabetes are risk factors for VBAC failure. Diabetic women could be at high risk of CS secondary to failed induction, labor arrest, and fetal distress [111]. Furthermore, pregnant women with diabetes are more likely to have increased BMI and weight gain, both of which have a negative influence on VBAC success. Prevent and control diabetes could help to increase the likelihood of VBAC.

HDCP has a negative impact on the VBAC success. HDCP could cause maternal vasospasm, which results in placental damage and relative insufficiency, leading to intrauterine fetal growth restriction (IUGR). These changes in vascular physiology could predispose women to a VBAC failure due to nonreassuring fetal status [70]. Fortunately, it was found that HDCP did not increase the risk of uterine rupture [70].

Our results show that interdelivery interval is not associated with a VBAC success. The interdelivery interval shorter than 24 months doesn’t relate to VBAC failure. However, there is only one study reporting the association between the interdelivery interval shorter than 18 months and the likelihood of VBAC [41], so we couldn’t conduct a meta-analysis to make sure whether the interval shorter than 18 months is a risk for failed VBAC.

Compared to spontaneous labor, induction of labor is more likely to decrease the likelihood of VBAC. However, an unfavorable cervix also decreases the chance of VBAC success. Whether to perform induction of labor and when to do it are the questions we meet in clinical practice. Our results showed that the gestational week at delivery is not associated to the VBAC success, whether the cut-off point was 37 gestational weeks, 40 gestational weeks or 41 gestational weeks. One recent study revealed that induction of labor at 39 gestational weeks could increase the chance of VBAC compared to expectant management [112], but also of uterine rupture. Thus, we should consider both benefits and harms to perform induction of labor for appropriate VBAC candidates. Gestational week might not serve as an argument for or against VBAC.

Smoke and epidural anesthesia are not associated with the chance of VBAC in our study. However, smoke could increase susceptibility for nonreassuring fetal heart rate, especially in the second stage of labor, leading to higher rate of instrumental delivery [113]. Pain relief during labor makes women more likely to choose TOLAC. However, epidural anesthesia could also relieve the pain caused by uterine rupture, which clinicians should be highly aware of.

During the prenatal counseling of women with one previous cesarean section, the probability of successful vaginal birth is one of the most crucial factors. The history of previous delivery (previous VB before CS, previous VBAC and the indications for previous CS), the characteristics of the pregnant women (age and race), the complications (obesity, diabetes and HDCP), the size of the fetus, the bishop score of the cervix, and the necessity of labor induction should be highly considered, which were associated to the success of VBAC. The clinicians should be also aware that uterine rupture could complicate with TOLAC. TOLAC under continuous monitoring by a skilled clinician and at facilities with 24-h surgery services [18] should be guaranteed to increase the safety of the delivery.

## Conclusions

Our results find that age, obesity, diabetes, HDCP, Bishop score, labor induction, birth weight, previous vaginal birth, and the indications for the previous CS should be considered as the factors related to the success of VBAC. This meta-analysis provides the most comprehensive review of previously reported maternal and fetal factors for the chance of VBAC. We believe that the results are important for women who are pregnant or are planning to become pregnant after a previous CS.

## Supplementary information

**Additional file 1: Figure S1.** Selection of studies (JPG 101 kb)

**Additional file 2: Figure S2.** Successful rates of VBAC among different continents. *P < 0.001; **P < 0.01; ***P < 0.001; ****P = 0.135 (JPG 56 kb)

**Additional file 3: Figure S3.** BMI associated with VBAC success - Standardized mean differences. (JPG 142 kb)

**Additional file 4: Figure S4.** BMI associated with VBAC success - Adjusted odds ratio. (JPG 114 kb)

**Additional file 5: Figure S5.** Association between Bishop score and VBAC. (A) Standardized mean differences; (B) Odds ratio; (C) Adjusted odds ratio. (JPG 114 kb)

**Additional file 6: Figure S6.** Birth weight associated with VBAC success. (A) Standardized mean differences; (B) Odds ratio; (C) Adjusted odds ratio. (JPG 171 kb)

**Additional file 7: Figure S7.** Previous vaginal birth before CS associated with VBAC success. (A) Odds ratio; (B) Adjusted odds ratio. (JPG 144 kb)

**Additional file 8: Figure S8.** Previous VBAC associated with VBAC success. (A) Odds ratio; (B) Adjusted odds ratio. (JPG 83 kb)

**Additional file 9: Figure S9.** Association between indication for previous CS and VBAC. (A) Association of suspected fetal macrosomia, failed induction, cephalopelvic disproportion, and hypertensive disorders complicating pregnancy as the indication for Previous CS with VBAC; (B) Dystocia or failure to progress as the indication for previous CS associated with VBAC success - odds ratio; (C) No association between fetal distress as the indication for previous CS and VBAC- odds ratio; (D) Fetal malpresentation as the indication for previous CS associated with VBAC success - odds ratio; (E) Dystocia or failure to progress as the indication for previous CS associated with VBAC success - adjusted odds ratio. (PNG 692 kb)

**Additional file 10: Figure S10.** Race associated with VBAC success. (A) White Race associated with VBAC success - odds ratio; (B) Non-White race associated with VBAC success - odds ratio;(C) Non-white Race associated with VBAC success - adjusted odds ratio. (JPG 164 kb)

**Additional file 11: Figure S11.** Association between age and VBAC. (A) Standardized mean differences; (B) Odds ratio; (C) Adjusted odds ratio. (JPG 169 kb)

**Additional file 12: Figure S12.** Hypertensive disorder complicating pregnancy associated with VBAC success - Adjusted odds ratio. (JPG 103 kb)

**Additional file 13: Figure S13.** Labor induction associated with VBAC success. (A) Odds ratio; (B) Adjusted odds ratio. (JPG 121 kb)

**Additional file 14: Figure S14.** No association between smoke and VBAC. (JPG 95 kb)

**Additional file 15: Figure S15.** No association between interdelivery interval and VBAC. (A) Standardized mean differences; (B) Odds ratio; (C) Adjusted odds ratio. (JPG 105 kb)

**Additional file 16: Figure S16.** No association between gestational week and VBAC. (A) Standardized mean differences; (B) Odds ratio; (C) Adjusted odds ratio. (JPG 164 kb)

**Additional file 17: Figure S17.** No association between epidural anesthesia and VBAC. (JPG 112 kb)

**Additional file 18: Table S1.** Characteristics of Meta analysis (DOCX 22 kb)

**Additional file 19: Table S2.** Characteristics of studies considered for this review. (DOCX 77 kb)

**Additional file 20: Table S3.** Newcastle-Ottawa quality assessment (DOCX 62 kb)

**Additional file 21: Table S4.**Subgroup analysis of factors (DOCX 73 kb)

**Additional file 22: Text S1**. MOOSE Checklist (DOC 106 kb)

**Additional file 23: Text S2.** PRISMA checklist (DOC 118 kb)

**Additional file 24: Appendix 1.** Search strategy. (DOCX 26 kb)

## Data Availability

All data analyzed during this study are included in this published article and its supplementary information files.

## Abbreviations

BMI: Body mass index
CI: Confidence interval
CPD: Cephalopelvic disproportion
CS: Cesarean section
HDCP: Hypertensive disorders complicating pregnancy
OR: Odds ratio
SMD: Standardized mean differences
TOLAC: Trial of labor after cesarean
VB: Vaginal birth
VBAC: Vaginal birth after cesarean section
WHO: World Health Organization
