# Supplementary figures and images for: Factors associated with successful vaginal birth after a cesarean section: a systematic review and meta-analysis
Source: BMC Pregnancy Childbirth. 2019 Oct 17;19:360. doi: 10.1186/s12884-019-2517-y (PMC6798397; doi:10.1186/s12884-019-2517-y)

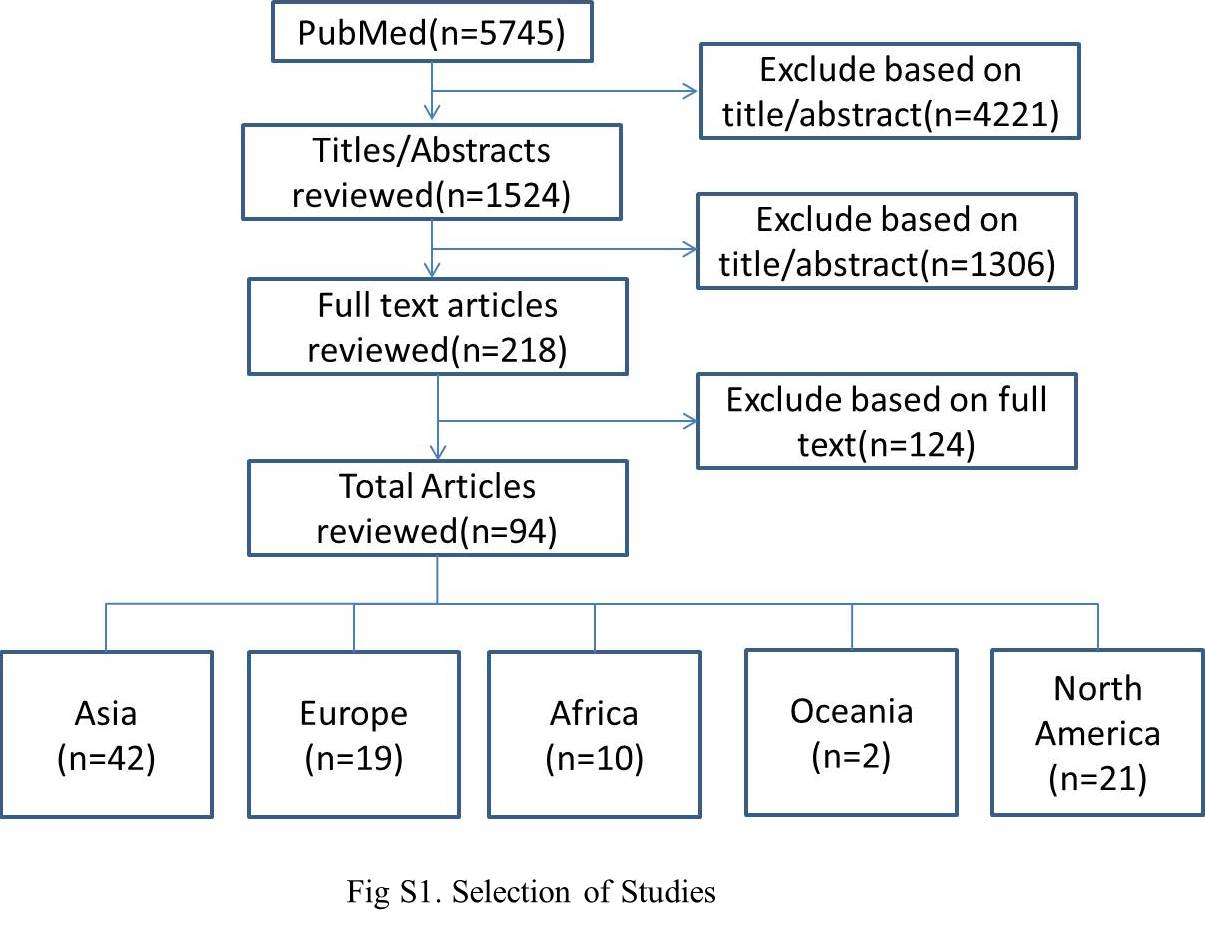

Supplement: Supplementary file 1 — Additional file 1: Figure S1. Selection of studies (JPG 101 kb) [file 12884_2019_2517_MOESM1_ESM.jpg]

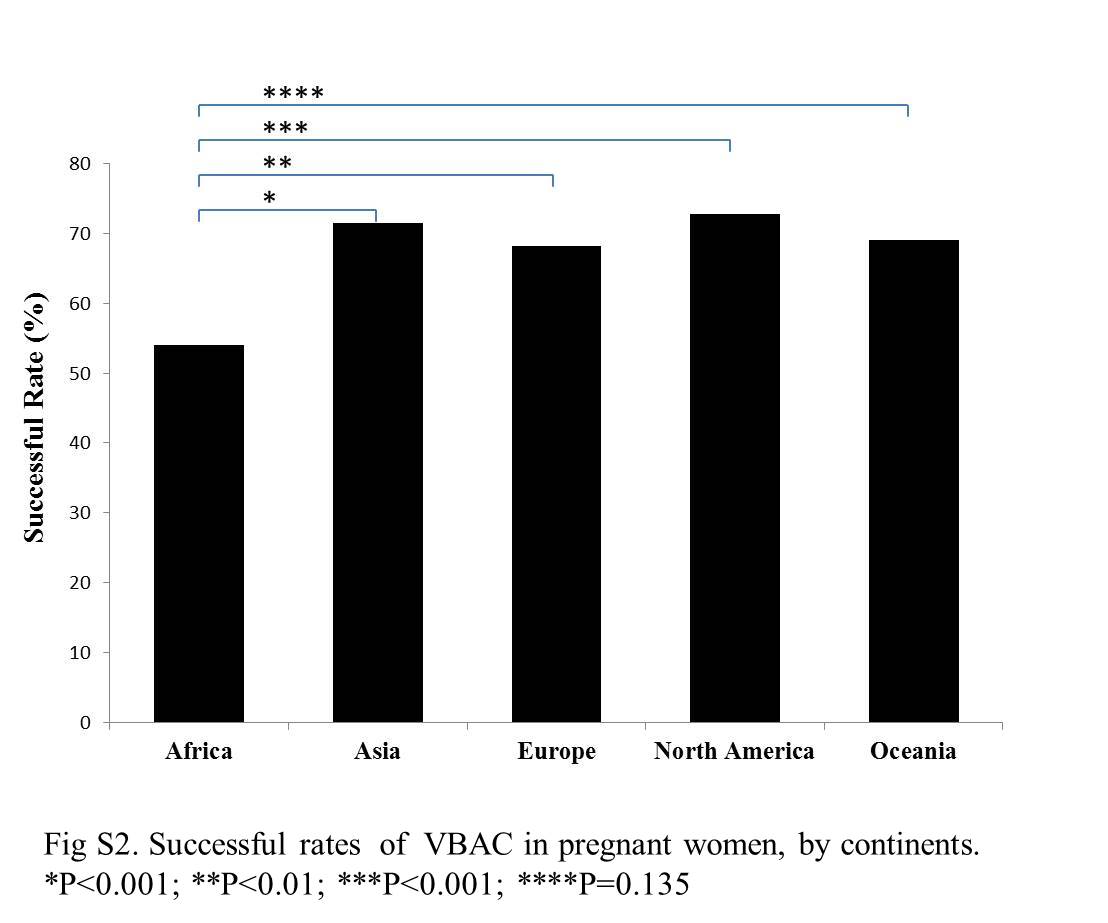

Supplement: Supplementary file 2 — Additional file 2: Figure S2. Successful rates of VBAC among different continents. *P < 0.001; **P < 0.01; ***P < 0.001; ****P = 0.135 (JPG 56 kb) [file 12884_2019_2517_MOESM2_ESM.jpg]

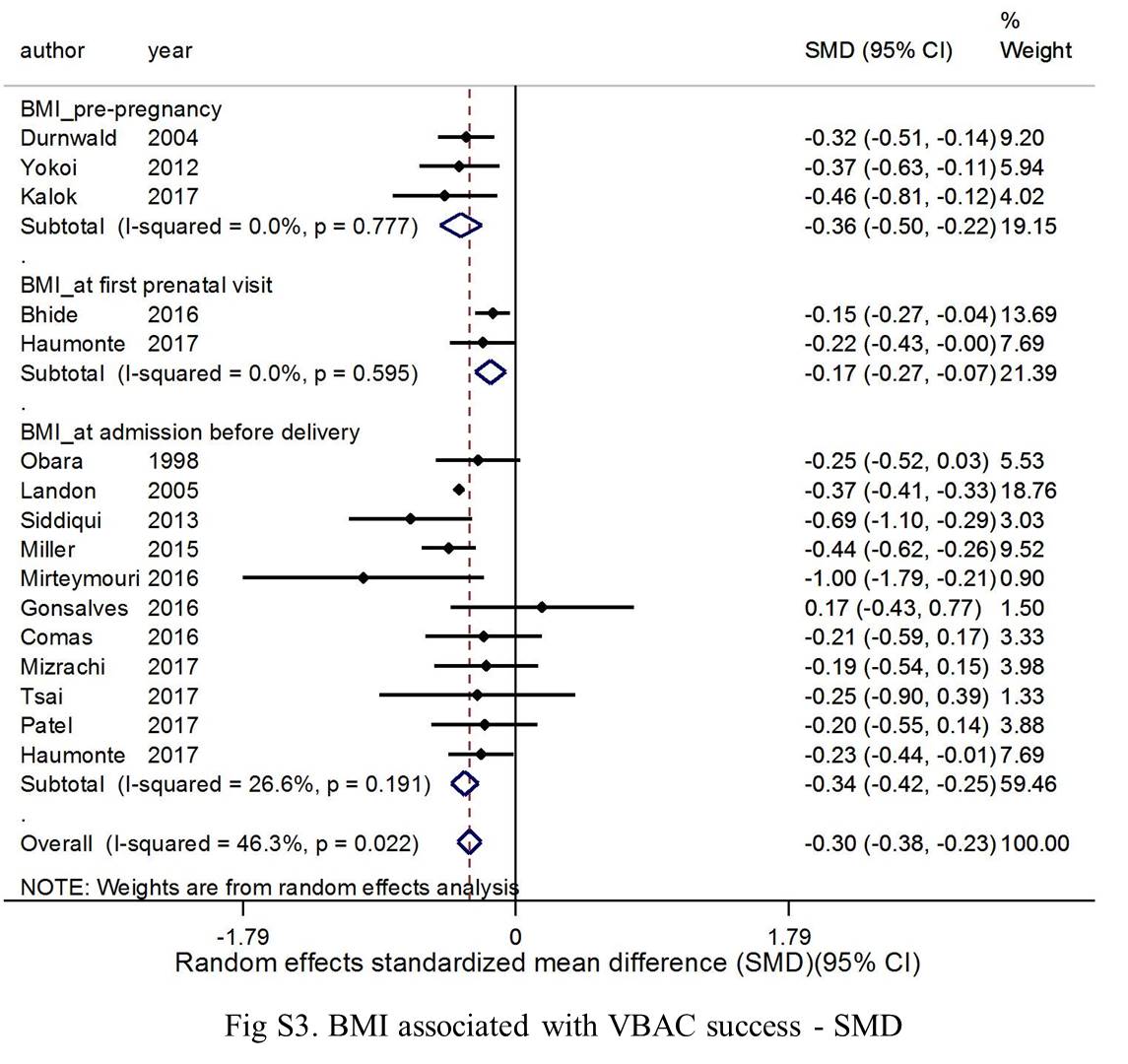

Supplement: Supplementary file 3 — Additional file 3: Figure S3. BMI associated with VBAC success - Standardized mean differences. (JPG 142 kb) [file 12884_2019_2517_MOESM3_ESM.jpg]

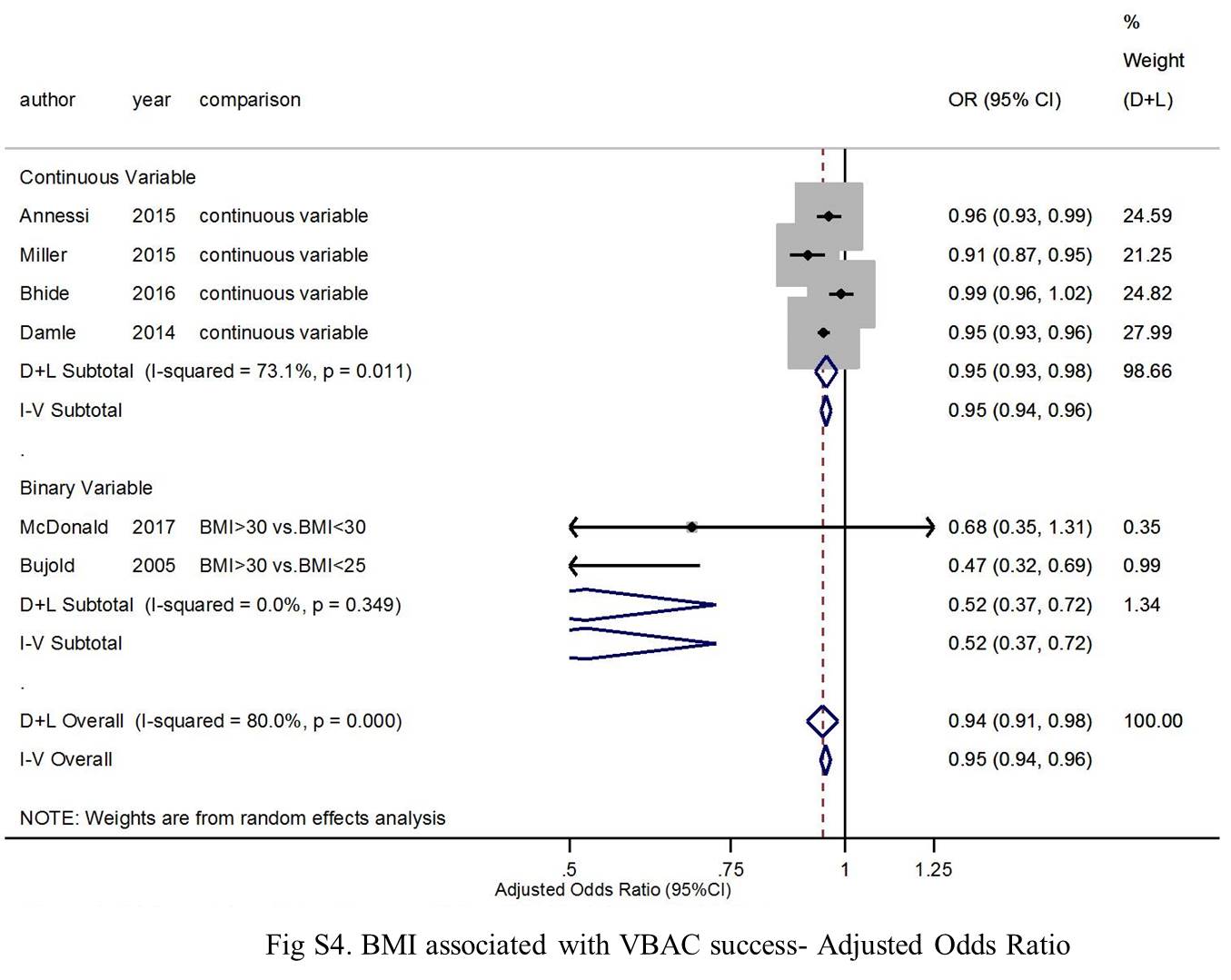

Supplement: Supplementary file 4 — Additional file 4: Figure S4. BMI associated with VBAC success - Adjusted odds ratio. (JPG 114 kb) [file 12884_2019_2517_MOESM4_ESM.jpg]

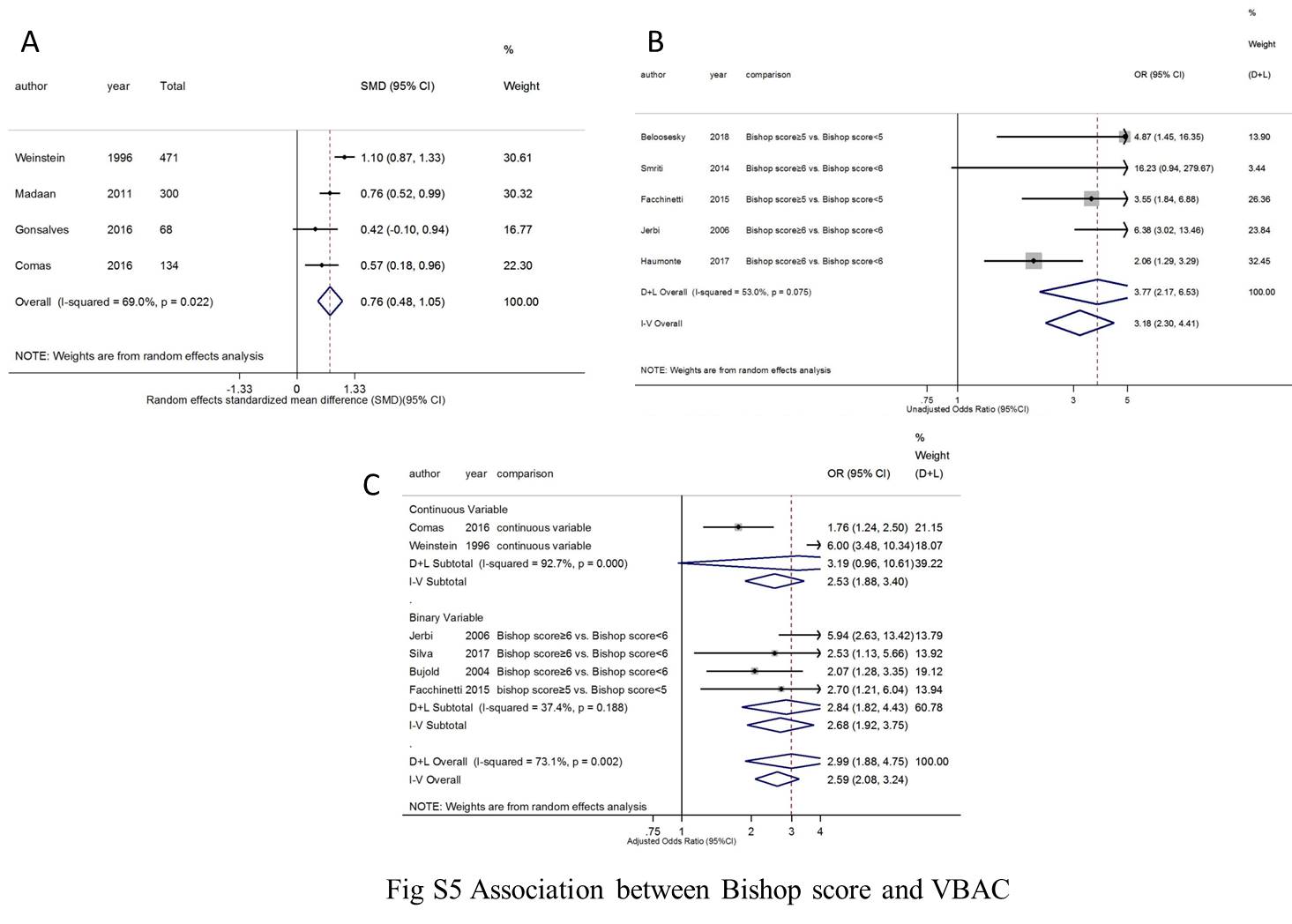

Supplement: Supplementary file 5 — Additional file 5: Figure S5. Association between Bishop score and VBAC. (A) Standardized mean differences; (B) Odds ratio; (C) Adjusted odds ratio. (JPG 114 kb) [file 12884_2019_2517_MOESM5_ESM.jpg]

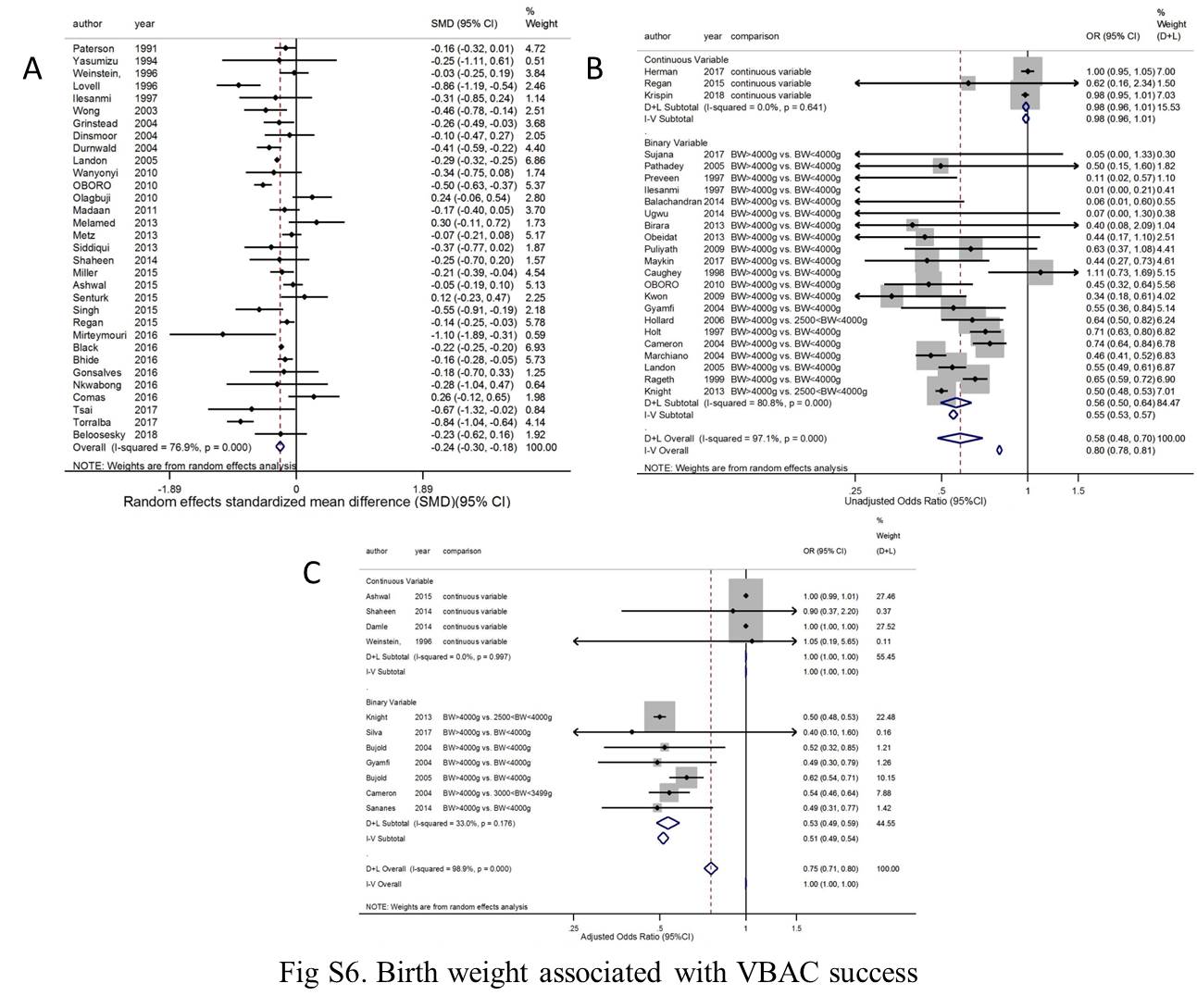

Supplement: Supplementary file 6 — Additional file 6: Figure S6. Birth weight associated with VBAC success. (A) Standardized mean differences; (B) Odds ratio; (C) Adjusted odds ratio. (JPG 171 kb) [file 12884_2019_2517_MOESM6_ESM.jpg]

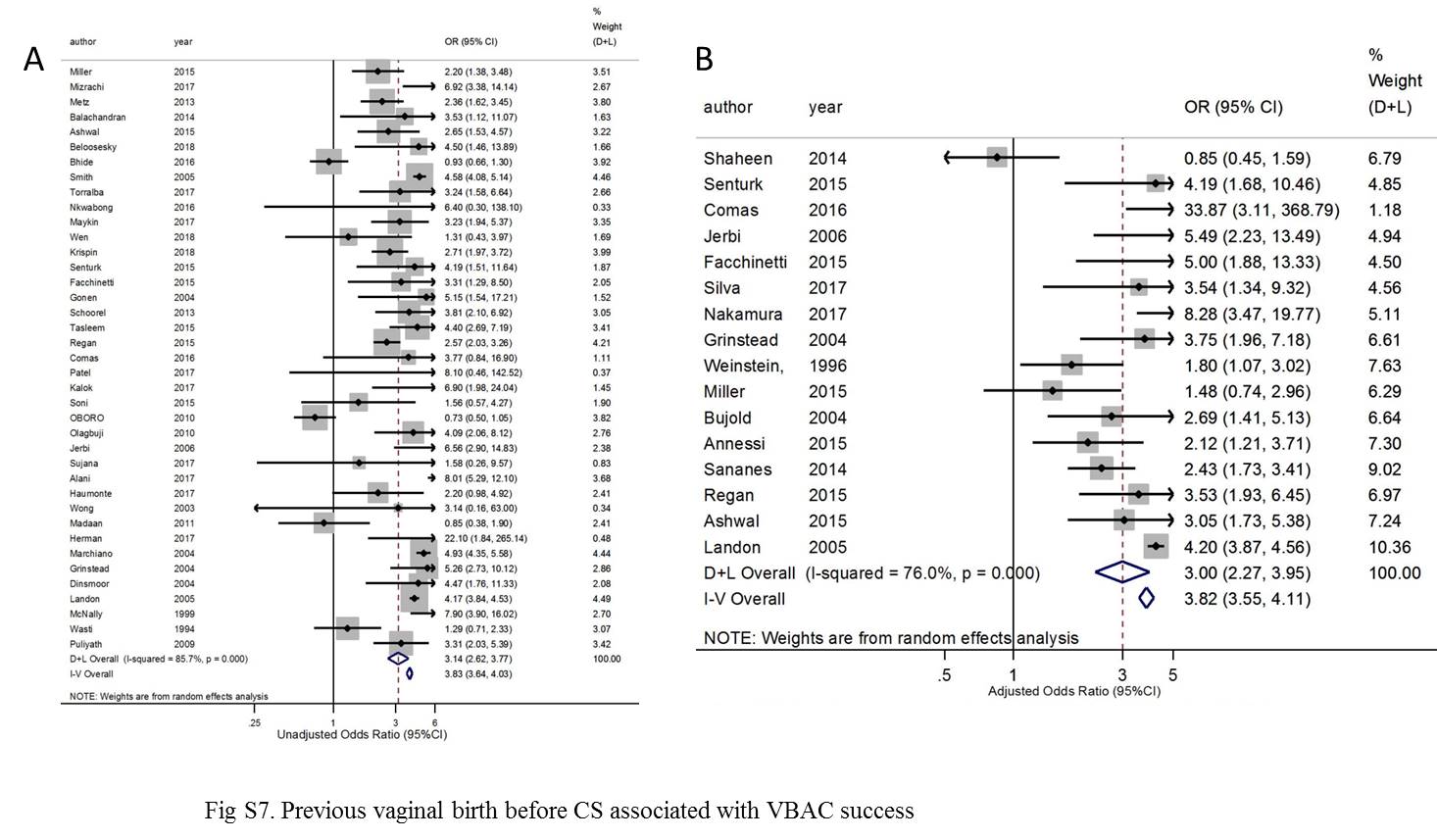

Supplement: Supplementary file 7 — Additional file 7: Figure S7. Previous vaginal birth before CS associated with VBAC success. (A) Odds ratio; (B) Adjusted odds ratio. (JPG 144 kb) [file 12884_2019_2517_MOESM7_ESM.jpg]

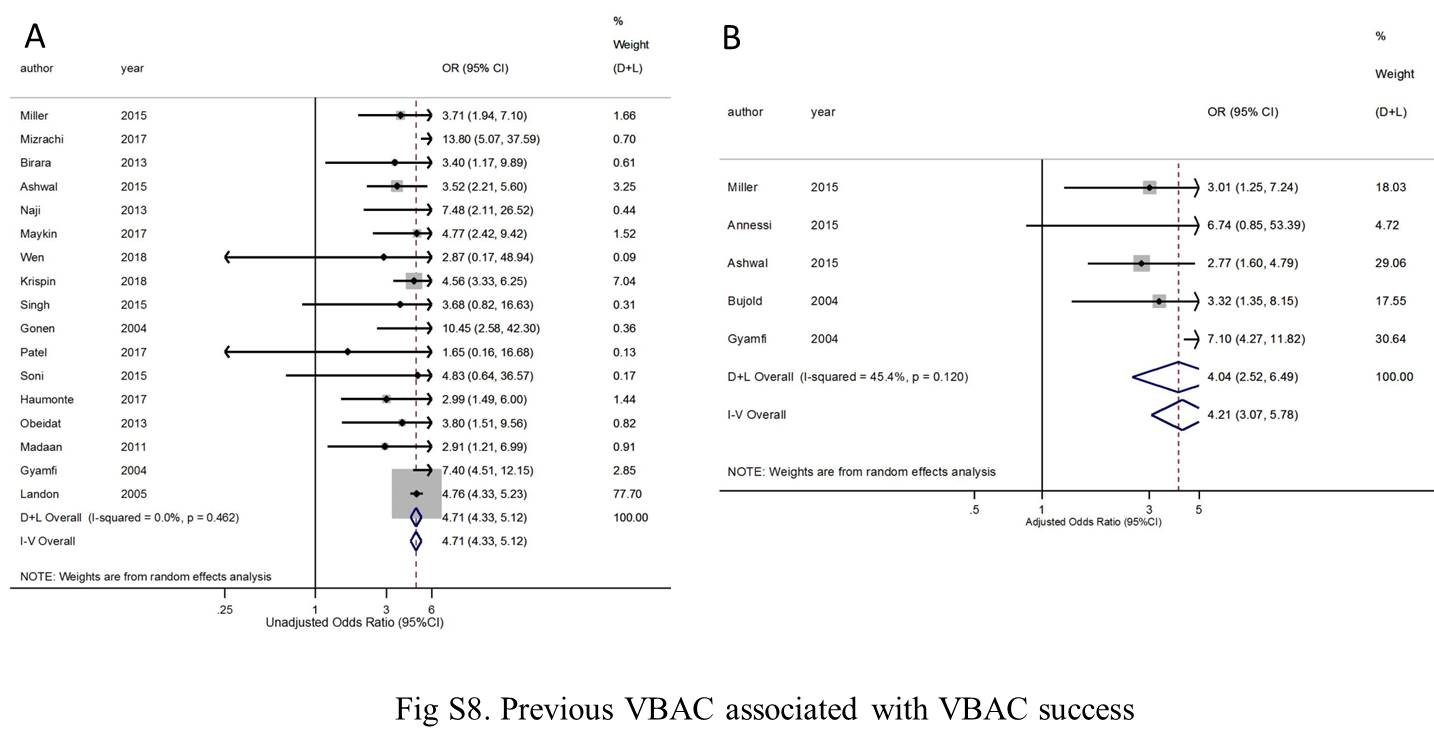

Supplement: Supplementary file 8 — Additional file 8: Figure S8. Previous VBAC associated with VBAC success. (A) Odds ratio; (B) Adjusted odds ratio. (JPG 83 kb) [file 12884_2019_2517_MOESM8_ESM.jpg]

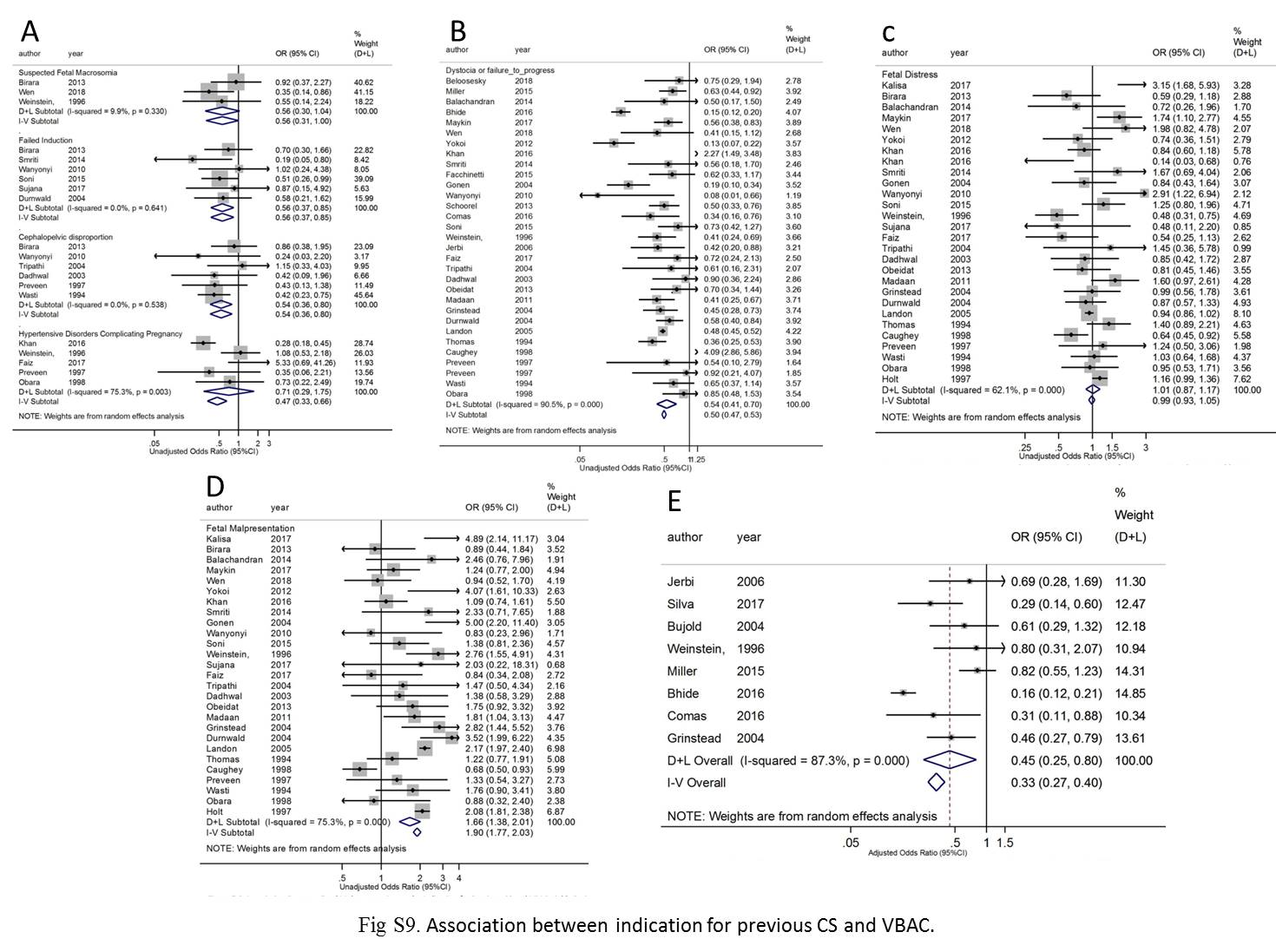

Supplement: Supplementary file 9 — Additional file 9: Figure S9. Association between indication for previous CS and VBAC. (A) Association of suspected fetal macrosomia, failed induction, cephalopelvic disproportion, and hypertensive disorders complicating pregnancy as the indication for Previous CS with VBAC; (B) Dystocia or failure to progress as the indication for previous CS associated with VBAC success - odds ratio; (C) No association between fetal distress as the indication for previous CS and VBAC- odds ratio; (D) Fetal malpresentation as the indication for previous CS associated with VBAC success - odds ratio; (E) Dystocia or failure to progress as the indication for previous CS associated with VBAC success - adjusted odds ratio. (PNG 692 kb) [file 12884_2019_2517_MOESM9_ESM.png]

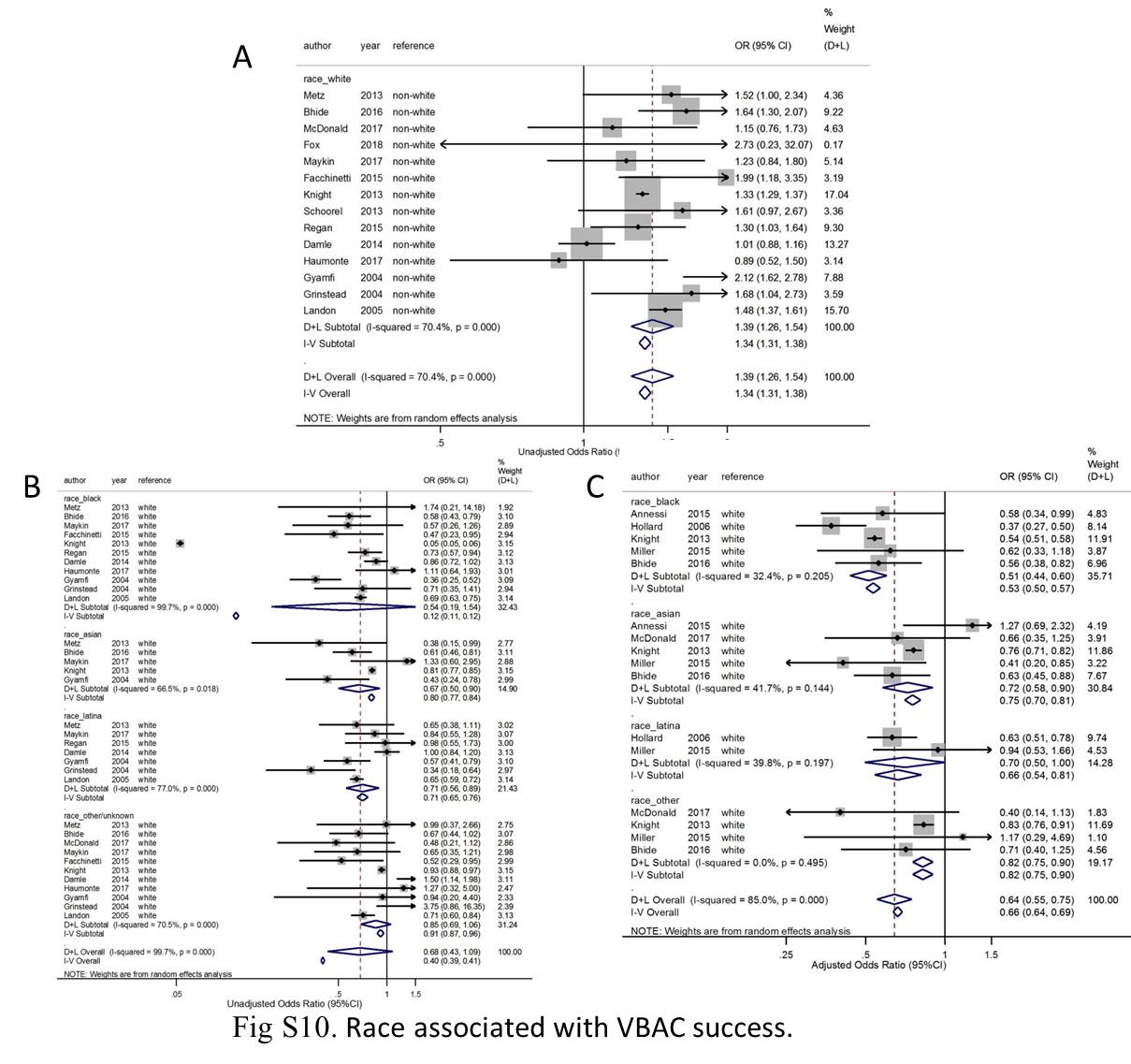

Supplement: Supplementary file 10 — Additional file 10: Figure S10. Race associated with VBAC success. (A) White Race associated with VBAC success - odds ratio; (B) Non-White race associated with VBAC success - odds ratio;(C) Non-white Race associated with VBAC success - adjusted odds ratio. (JPG 164 kb) [file 12884_2019_2517_MOESM10_ESM.jpg]

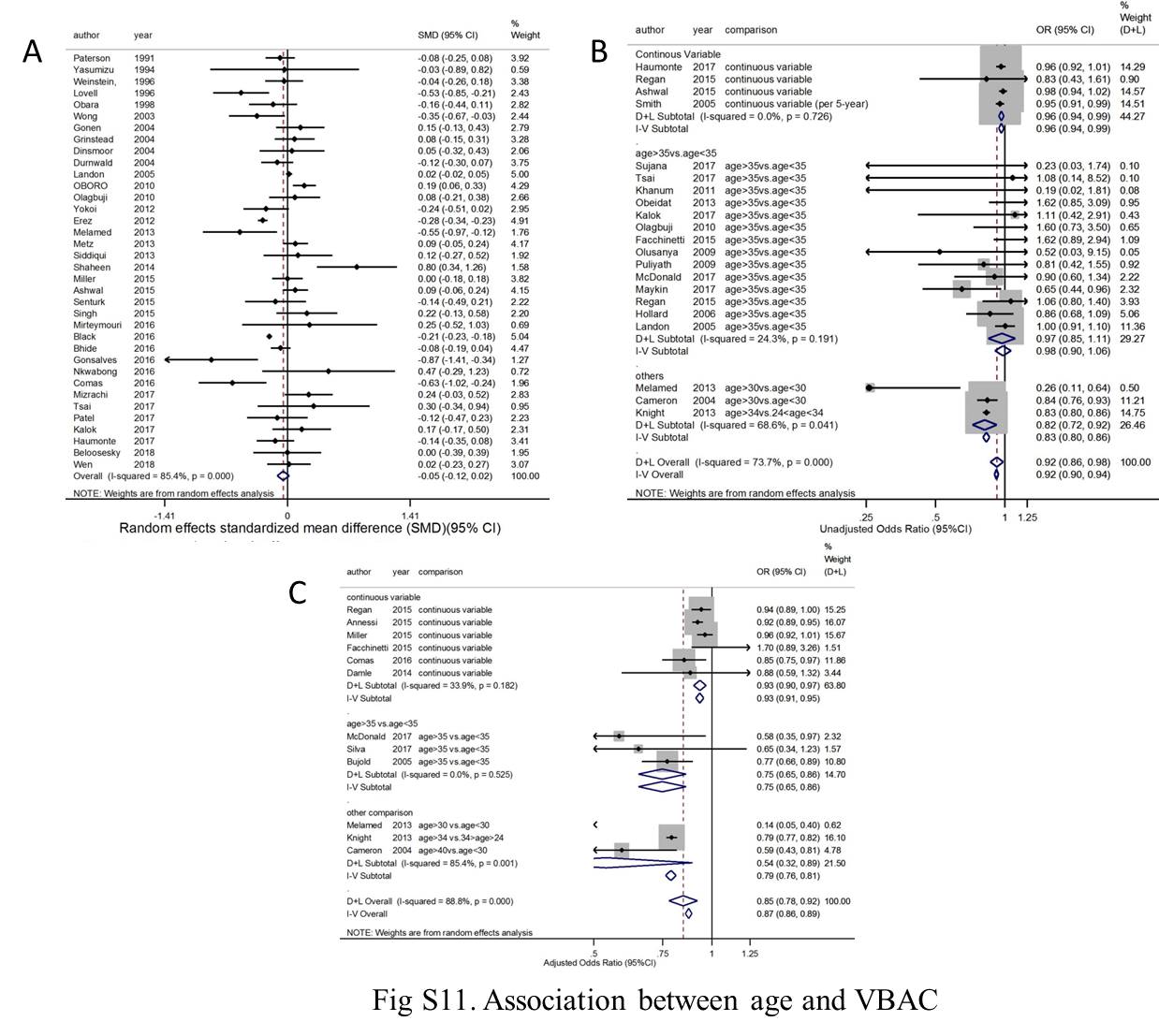

Supplement: Supplementary file 11 — Additional file 11: Figure S11. Association between age and VBAC. (A) Standardized mean differences; (B) Odds ratio; (C) Adjusted odds ratio. (JPG 169 kb) [file 12884_2019_2517_MOESM11_ESM.jpg]

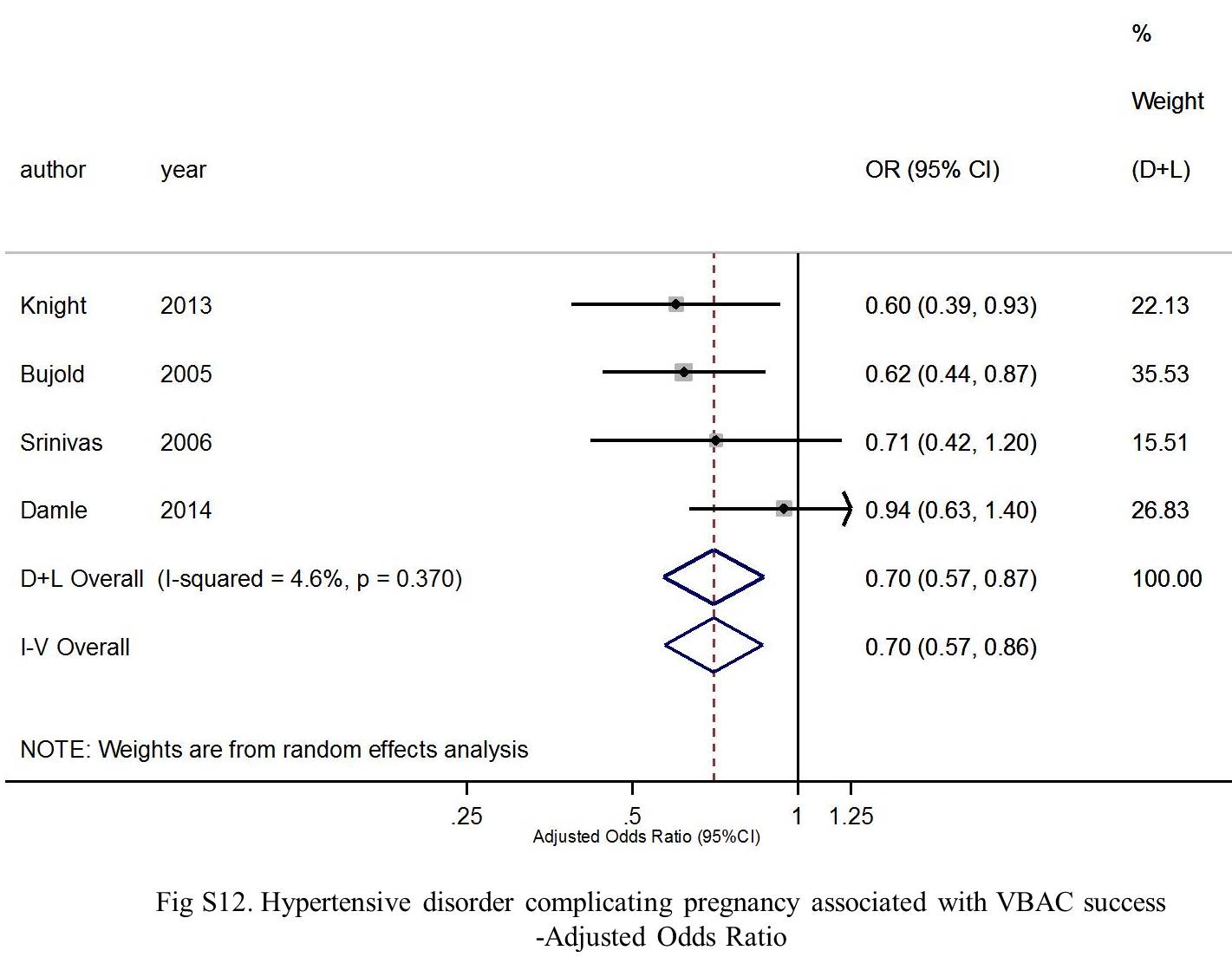

Supplement: Supplementary file 12 — Additional file 12: Figure S12. Hypertensive disorder complicating pregnancy associated with VBAC success - Adjusted odds ratio. (JPG 103 kb) [file 12884_2019_2517_MOESM12_ESM.jpg]

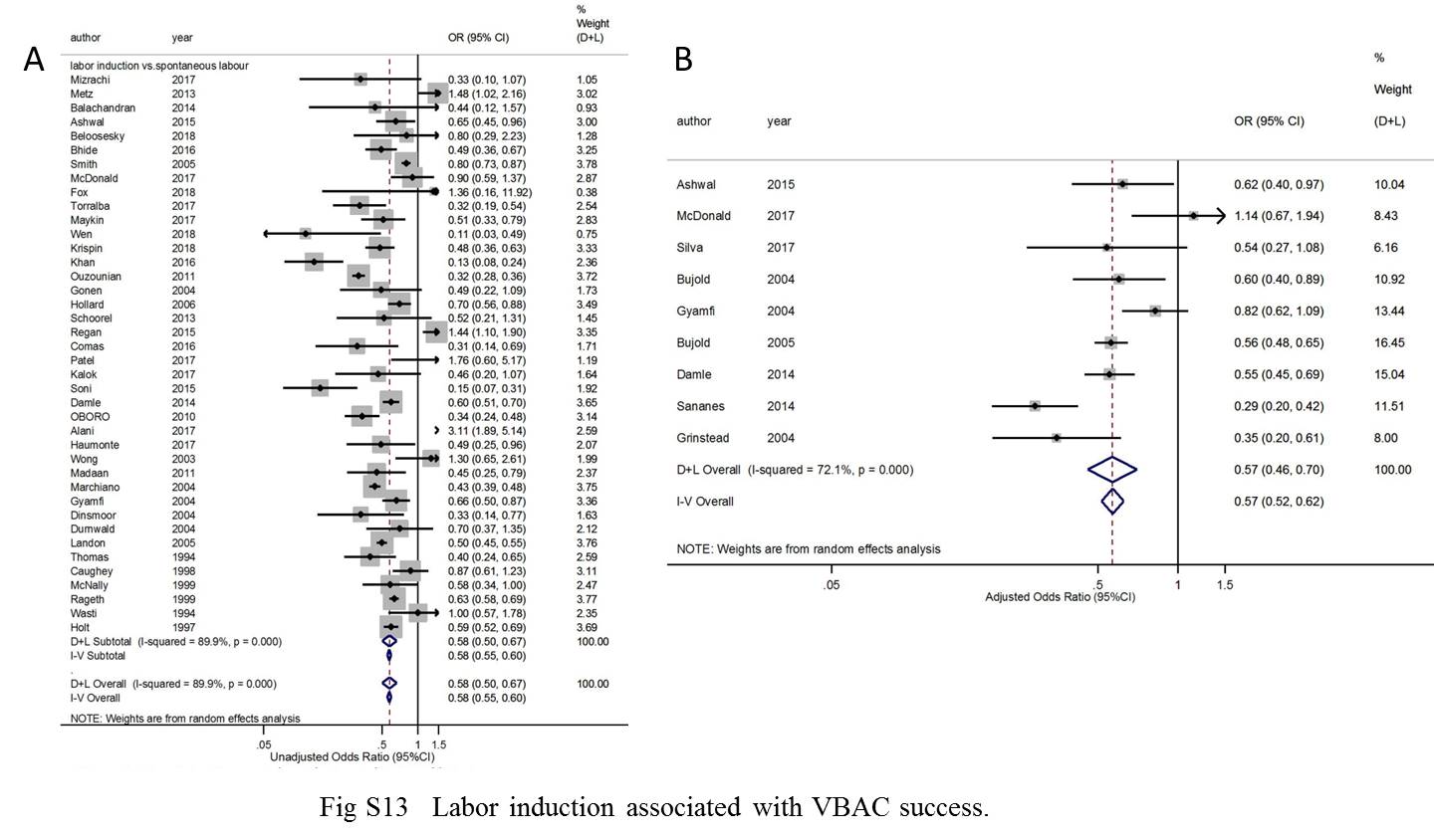

Supplement: Supplementary file 13 — Additional file 13: Figure S13. Labor induction associated with VBAC success. (A) Odds ratio; (B) Adjusted odds ratio. (JPG 121 kb) [file 12884_2019_2517_MOESM13_ESM.jpg]

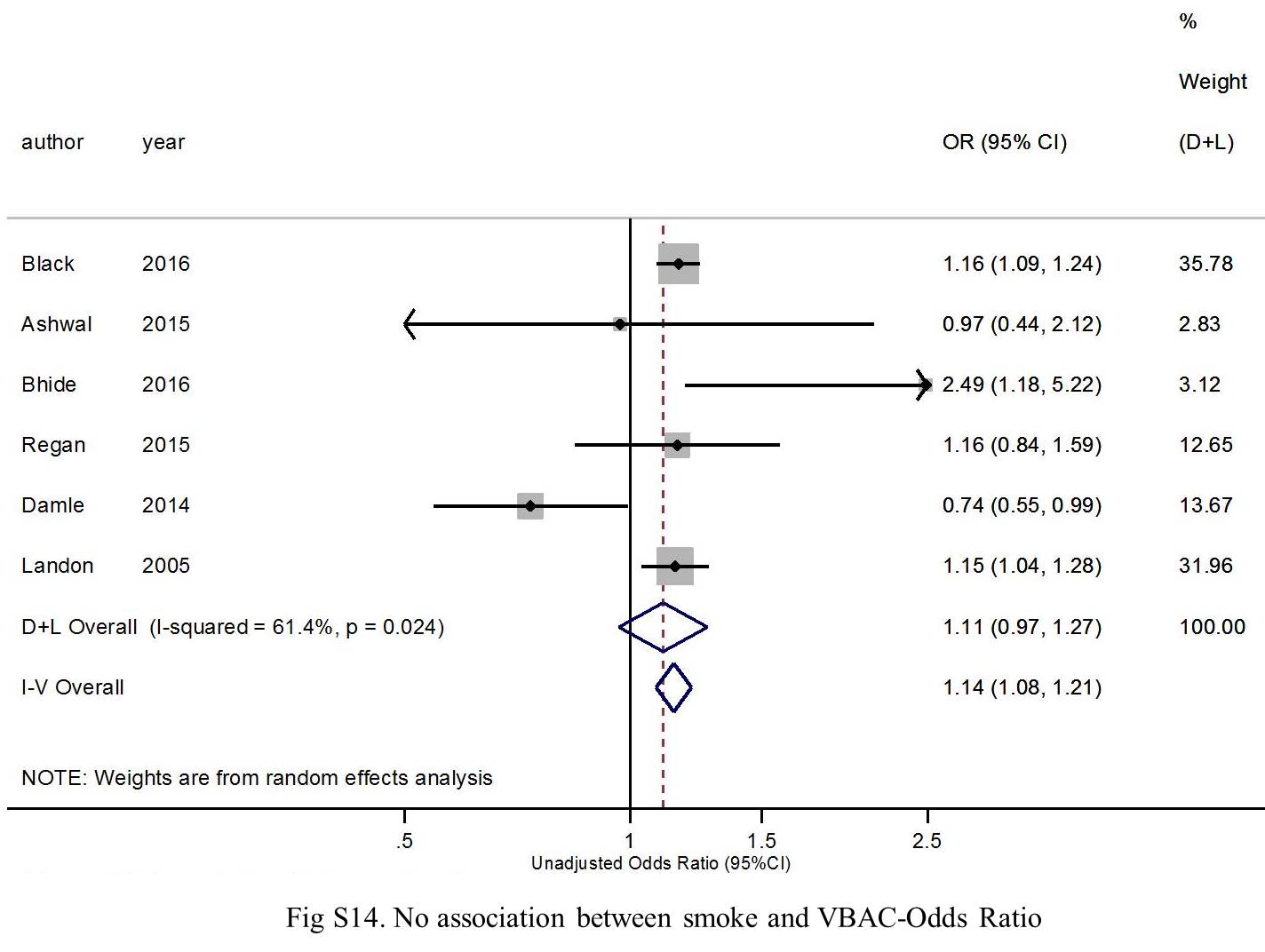

Supplement: Supplementary file 14 — Additional file 14: Figure S14. No association between smoke and VBAC. (JPG 95 kb) [file 12884_2019_2517_MOESM14_ESM.jpg]

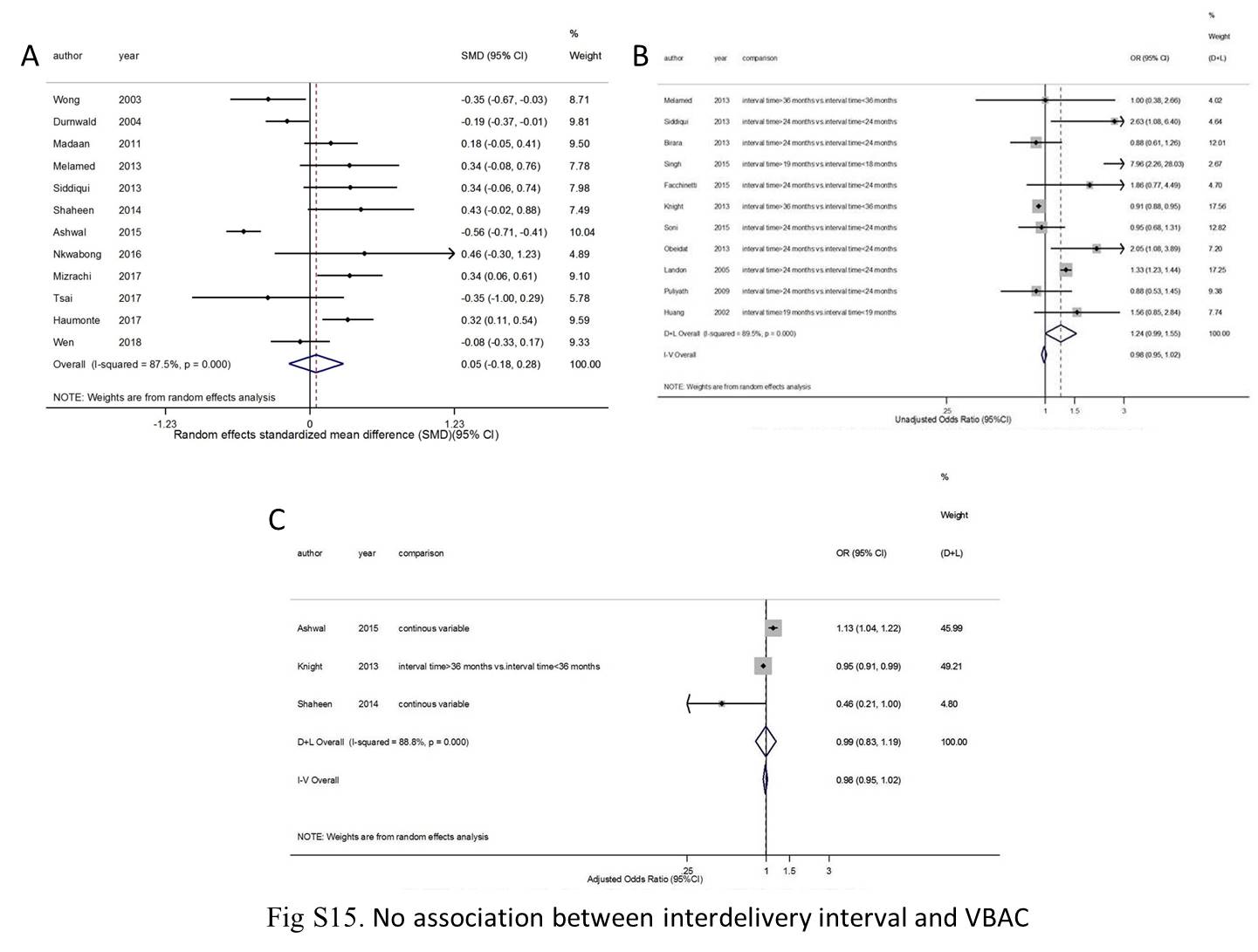

Supplement: Supplementary file 15 — Additional file 15: Figure S15. No association between interdelivery interval and VBAC. (A) Standardized mean differences; (B) Odds ratio; (C) Adjusted odds ratio. (JPG 105 kb) [file 12884_2019_2517_MOESM15_ESM.jpg]

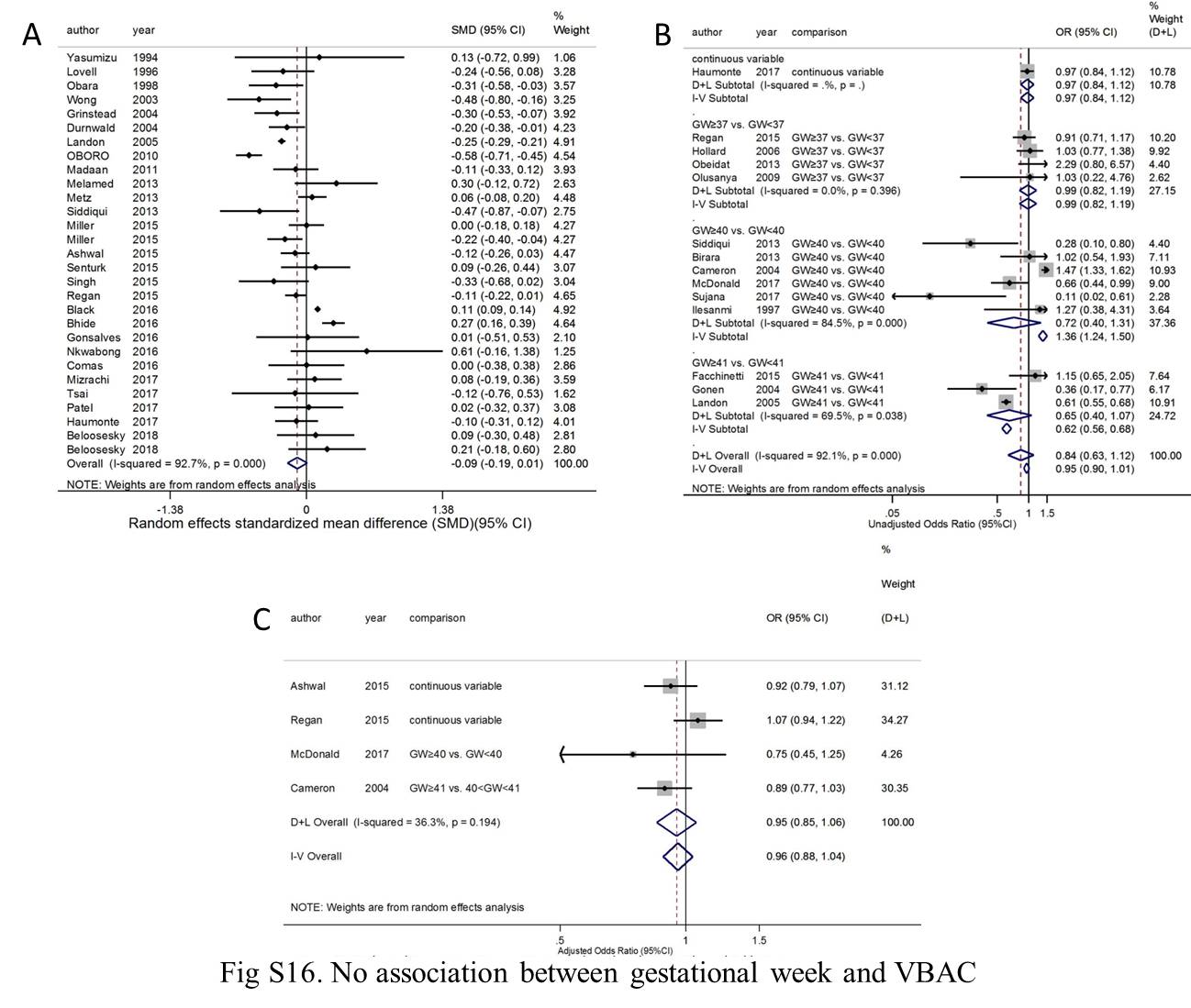

Supplement: Supplementary file 16 — Additional file 16: Figure S16. No association between gestational week and VBAC. (A) Standardized mean differences; (B) Odds ratio; (C) Adjusted odds ratio. (JPG 164 kb) [file 12884_2019_2517_MOESM16_ESM.jpg]

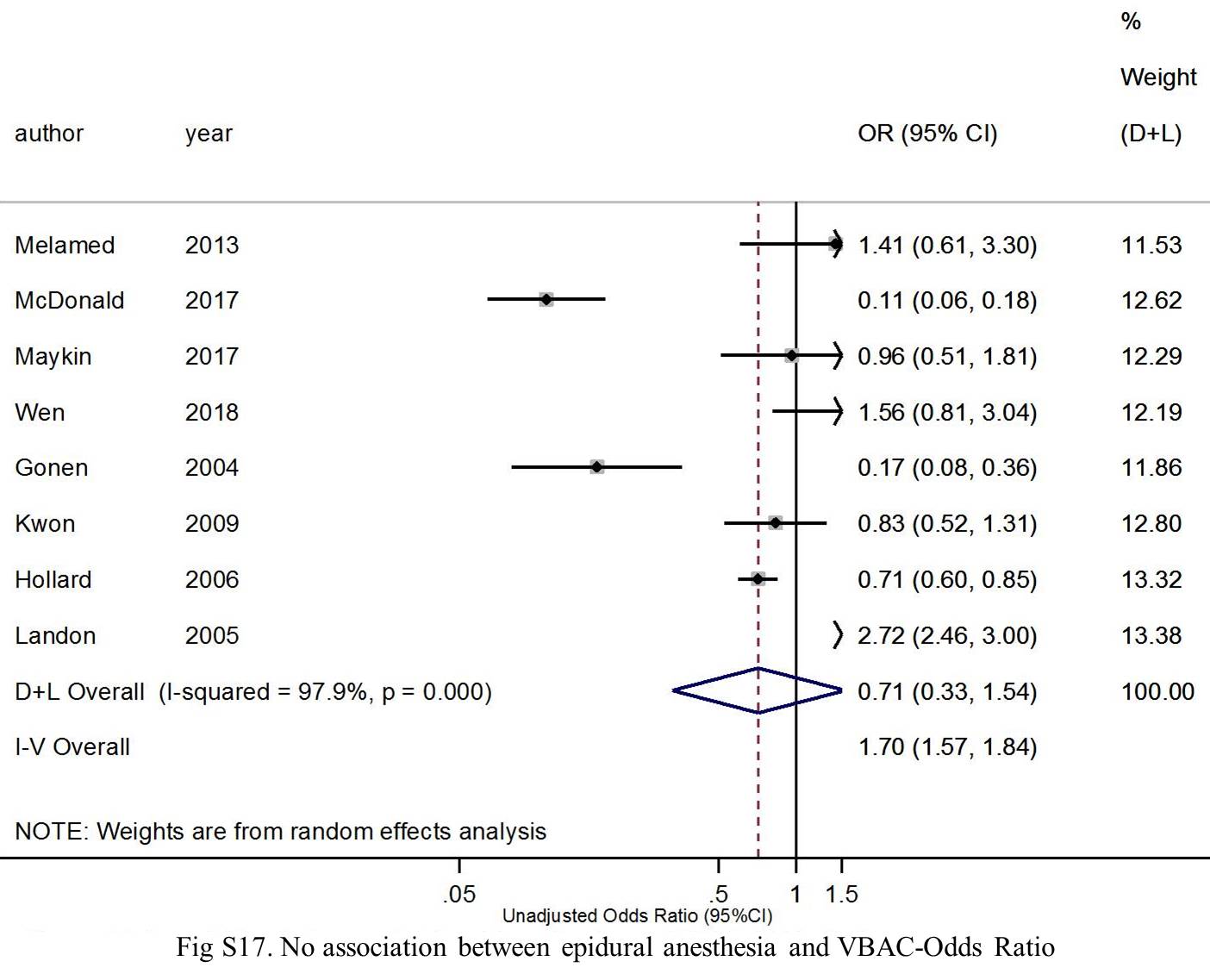

Supplement: Supplementary file 17 — Additional file 17: Figure S17. No association between epidural anesthesia and VBAC. (JPG 112 kb) [file 12884_2019_2517_MOESM17_ESM.jpg]
