# Supplementary material for: Factors associated with successful vaginal birth after a cesarean section: a systematic review and meta-analysis
Source: BMC Pregnancy Childbirth. 2019 Oct 17;19:360. doi: 10.1186/s12884-019-2517-y (PMC6798397; doi:10.1186/s12884-019-2517-y)
Supplement: Supplementary file 18 — Additional file 18: Table S1. Characteristics of Meta analysis (DOCX 22 kb) [file 12884_2019_2517_MOESM18_ESM.docx]

Table S1 Characteristics of the meta-analysis

| Item | Content |
| --- | --- |
| Countries | 34 |
| Continents | 5 |
| Total number of individuals | 239,006 |
| Total number of articles | 94 |
| Age (Case Mean) | 24-35 |
| Age (Control Mean) | 25-35 |
| Maternal and fetal factors | Age, BMI, smoke, diabetes, hypertension, prior VB before CS, prior VBAC, interdelivery interval, indication for prior CS, race, gestational weeks, Bishop score, birth weight, epidural anesthesia, labor induction |

BMI: body mass index; VB: vaginal birth; VBAC: vaginal birth after cesarean; CS: cesarean section.
