# Supplementary material for: Factors associated with successful vaginal birth after a cesarean section: a systematic review and meta-analysis
Source: BMC Pregnancy Childbirth. 2019 Oct 17;19:360. doi: 10.1186/s12884-019-2517-y (PMC6798397; doi:10.1186/s12884-019-2517-y)
Supplement: Supplementary file 19 — Additional file 19: Table S2. Characteristics of studies considered for this review. (DOCX 77 kb) [file 12884_2019_2517_MOESM19_ESM.docx]

Table S2. Characteristics of studies included in the meta-analyses

| Author | Year | Country | Design | N Total | N Case | Age | BMI | Smoke | Diabetes | Hypertension | Prior VB before CS | Prior VBAC | Interdelivery interval | Indication for prior CS | Race/ethnicity | Gestational weeks | Bishop score | Birth weight | Epidural anesthesia | Labor induction |
| --- | --- | --- | --- | --- | --- | --- | --- | --- | --- | --- | --- | --- | --- | --- | --- | --- | --- | --- | --- | --- |
| Miller | 2015 | USA | cohort | 678 | 517 | × | × |  |  |  | × | × |  | × | × | × |  | × |  |  |
| Mirteymouri | 2016 | Iran | cross sectional | 80 | 73 | × | × |  |  |  | × |  |  |  |  |  |  | × |  |  |
| Kalisa | 2017 | Rwanda | cohort | 297 | 134 |  |  |  |  |  |  |  |  | × |  |  |  |  |  |  |
| Melamed | 2013 | Israel | cohort | 93 | 57 | × |  |  | × | × | × |  | × |  |  | × |  | × | × | × |
| Mizrachi | 2017 | Israel | cohort | 231 | 155 | × | × |  | × | × | × | × | × |  | × | × |  |  |  | × |
| Metz | 2013 | USA | cohort | 1170 | 938 | × | × |  | × | × | × |  |  |  | × | × |  | × |  | × |
| Tsai | 2017 | China | cohort | 73 | 62 | × | × |  | × | × | × |  | × |  |  | × |  | × |  |  |
| Siddiqui | 2013 | Pakistan | cross sectional | 122 | 88 | × | × |  |  |  |  |  | × |  |  | × |  | × |  | × |
| Birara | 2013 | Ethiopia | case control | 204 | 101 | × |  |  |  |  |  | × | × | × |  | × |  | × |  |  |
| Black | 2016 | UK | cohort | 22226 | 13379 | × |  | × | × |  |  |  |  |  |  | × |  | × |  |  |
| Balachandran | 2014 | UAE | cohort | 115 | 96 |  |  |  |  |  | × |  |  | × |  |  |  | × |  | × |
| Annessi | 2016 | Italy | cohort | 1100 | 857 | × | × |  |  |  | × | × |  |  | × |  |  |  |  | × |
| Cameron | 2004 | Australia | cohort | 6983 | 4542 | × |  |  |  |  |  |  |  |  |  | × |  | × |  |  |
| Ashwal | 2015 | Israel | cohort | 1767 | 1563 | × |  | × |  |  | × | × | × | × |  | × |  | × |  | × |
| Beloosesky | 2018 | Israel | cohort | 105 | 63 | × | × |  |  |  | × |  |  | × |  | × | × | × |  | × |
| Bhide | 2016 | UK | cohort | 1463 | 1050 | × | × | × | × |  | × |  |  | × | × | × |  | × |  | × |
| Smith | 2005 | UK | cohort | 11643 | 8576 | × |  |  |  |  | × |  |  |  |  | × |  |  |  | × |
| Naji | 2013 | UK | cohort | 121 | 74 |  |  |  |  |  |  | × |  |  |  |  |  |  |  | × |
| McDonald | 2017 | New Zealand | cohort | 534 | 391 | × | × |  |  |  |  |  |  | × | × | × |  |  | × | × |
| Fox | 2018 | USA | cohort | 100 | 84 |  |  |  |  |  | × |  |  |  | × |  |  |  |  | × |
| Torralba | 2017 | Spain | cohort | 418 | 215 |  |  |  |  |  | × |  |  |  |  |  |  | × |  | × |
| Gonsalves | 2016 | Oman | cohort | 68 | 47 | × | × |  |  |  | × |  |  |  |  | × | × | × |  |  |
| Nkwabong | 2016 | Cameroon | cohort | 36 | 27 | × |  |  |  |  | × | × | × |  |  | × |  | × |  |  |
| Maykin | 2017 | USA | cohort | 568 | 402 | × | × |  | × |  | × | × |  | × | × |  |  | × | × | × |
| Wen | 2018 | China | cohort | 444 | 370 | × |  |  |  | × | × | × | × | × |  |  |  |  | × | × |
| Yokoi | 2012 | Japan | cohort | 725 | 664 | × | × |  |  |  | × |  |  | × |  |  |  |  |  |  |
| Krispin | 2018 | Israel | cohort | 3256 | 2900 |  |  |  |  |  | × | × |  |  |  |  |  | × |  | × |
| Khan | 2016 | Abbottabad-Pakistan | cross sectional | 764 | 535 |  |  |  |  |  |  |  |  | × |  |  |  |  |  | × |
| Senturk | 2015 | Turkey | cohort | 127 | 70 | × |  |  |  |  | × |  |  |  |  | × |  | × |  |  |
| Singh | 2015 | India | cohort | 142 | 96 | × |  |  |  |  |  | × | × |  |  | × |  | × |  |  |
| Smriti | 2014 | India | cohort | 100 | 65 |  | × |  |  |  |  |  |  | × |  |  | × |  |  |  |
| Facchinetti | 2015 | Italy | cohort | 234 | 120 | × | × |  |  |  | × |  | × | × | × | × | × |  |  |  |
| Erez | 2012 | Italy | cohort | 5519 | 3622 | × |  |  | × | × |  |  |  |  |  |  |  |  |  |  |
| Knight | 2013 | UK | cohort | 75086 | 47602 | × |  |  | × | × |  |  | × |  | × |  |  | × |  |  |
| Ouzounian | 2011 | USA | cross sectional | 6833 | 5531 |  |  |  |  |  |  |  |  |  |  |  |  |  |  | × |
| Gonen | 2004 | Israel | cohort | 339 | 279 | × |  |  |  |  | × | × |  | × |  | × |  |  | × | × |
| Kwon | 2009 | Korean | cohort | 1148 | 956 |  |  |  |  |  |  |  |  | × |  |  |  | × | × |  |
| Hollard | 2006 | USA | cohort | 2575 | 2000 | × | × |  | × | × |  |  |  |  | × | × |  | × | × | × |
| Wanyonyi | 2010 | Kenya | cohort | 89 | 44 |  |  |  |  |  |  |  |  | × |  |  |  | × |  |  |
| Ugwu | 2014 | Nigeria | cohort | 180 | 90 |  |  |  |  |  |  |  |  |  |  |  |  | × |  |  |
| Schoorel | 2013 | Netherlands | cohort | 515 | 371 |  | × |  |  |  | × |  |  | × | × |  |  |  |  | × |
| Tasleem | 2015 | Pakistan | cohort | 592 | 418 |  | × |  |  |  | × |  |  |  |  |  |  |  |  |  |
| Regan | 2015 | USA | cohort | 1433 | 974 | × |  | × |  |  | × |  |  |  | × | × |  | × |  | × |
| Paterson | 1991 | UK | cohort | 664 | 471 | × |  |  |  |  |  |  |  |  |  |  |  | × |  |  |
| Comas | 2016 | Spain | cohort | 134 | 98 | × | × |  | × | × | × |  |  | × |  | × | × | × |  | × |
| Patel | 2017 | India | cohort | 139 | 90 | × | × |  |  | × | × | × |  |  |  | × |  |  |  | × |
| Nakamura | 2017 | Japan | cohort | 333 | 242 | × |  |  |  |  | × |  |  | × |  |  |  |  |  |  |
| Kalok | 2017 | Malaysia | cohort | 186 | 142 | × | × |  |  |  | × |  |  | × | × |  |  |  |  | × |
| Soni | 2015 | India | cohort | 482 | 383 |  |  |  |  |  | × | × | × | × |  |  |  | × |  | × |
| Shaheen | 2014 | Pakistan | cross sectional | 95 | 68 | × |  |  |  |  | × | × | × |  |  |  |  | × |  |  |
| Damle | 2014 | USA | cohort | 3291 | 1604 | × | × | × | × | × |  |  |  |  | × |  |  | × |  | × |
| Sananes | 2014 | France | cohort | 1269 | 958 |  |  |  |  |  | × |  |  |  |  |  |  | × |  | × |
| OBORO | 2010 | Nigeria | cohort | 1013 | 683 | × |  |  |  |  | × |  |  |  |  | × |  | × |  | × |
| Olagbuji | 2010 | Nigeria | cohort | 188 | 72 | × |  |  |  |  | × |  |  | × |  |  |  | × |  |  |
| Weinstein | 1996 | Israel | cohort | 471 | 368 | × |  |  |  |  | × |  |  | × |  |  | × | × |  |  |
| Pathadey | 2005 | UK | cohort | 78 | 59 |  |  |  |  |  |  |  |  |  |  |  |  | × |  |  |
| Jerbi | 2006 | Tunisia | cohort | 173 | 129 |  |  |  |  |  | × |  |  | × |  |  | × |  |  |  |
| Sujana | 2017 | Indonesia | cohort | 57 | 49 | × | × |  |  |  | × |  |  | × |  | × |  | × |  |  |
| Silva | 2017 | Portugal | cohort | 292 | 134 | × |  |  |  |  | × |  |  | × |  |  | × | × |  | × |
| Faiz | 2017 | Saudi Arabia | cohort | 181 | 143 |  |  |  |  |  |  |  |  | × |  |  |  |  |  |  |
| Alani | 2017 | Bahrain | cohort | 568 | 236 |  |  |  |  |  | × |  |  | × |  |  |  |  |  | × |
| Haumonte | 2017 | France | cohort | 367 | 240 | × | × |  |  | × | × | × | × | × | × | × | × |  |  | × |
| Puri | 2011 | India | cohort | 205 | 72 |  |  |  |  |  |  |  |  | × |  |  |  |  |  |  |
| Tripathi | 2004 | India | cohort | 81 | 59 |  |  |  |  |  |  |  |  | × |  |  |  |  |  |  |
| Dadhwal | 2003 | India | cohort | 154 | 97 |  |  |  |  |  |  |  |  | × |  |  |  |  |  |  |
| Obeidat | 2013 | Jordan | cohort | 207 | 117 | × |  |  |  |  |  | × | × | × |  | × |  | × |  |  |
| Wong | 2003 | China | cohort | 170 | 113 | × |  |  |  |  | × |  | × |  |  | × |  | × |  | × |
| Madaan | 2011 | India | cohort | 300 | 161 |  |  |  |  |  | × | × | × | × |  | × | × | × |  | × |
| Herman | 2017 | Israel | case control | 66 | 47 |  | × |  | × |  | × |  |  |  |  |  |  | × |  |  |
| Khanum | 2011 | Pakistan | cohort | 92 | 64 | × |  |  |  |  |  |  |  |  |  |  |  |  |  |  |
| Marchiano | 2004 | USA | cohort | 9860 | 7320 |  |  |  | × |  | × |  |  | × |  |  |  | × |  | × |
| Olusanya | 2009 | Nigeria | cross sectional | 264 | 15 | × |  |  |  | × |  |  |  |  |  | × |  | × |  |  |
| Bujold | 2004 | Canada | cohort | 685 | 474 |  |  |  |  |  | × | × |  | × |  |  | × | × |  | × |
| Gyamfi | 2004 | USA | cohort | 1216 | 938 |  |  |  | × |  |  | × |  | × | × |  |  | × |  | × |
| Grinstead | 2004 | USA | cohort | 429 | 334 | × |  |  | × | × | × |  |  | × | × | × |  | × |  | × |
| Dinsmoor | 2004 | USA | cohort | 153 | 117 | × |  |  |  |  | × |  |  |  | × |  |  | × |  | × |
| Durnwald | 2004 | USA | cohort | 510 | 337 | × | × |  | × |  |  |  | × | × | × | × |  | × |  | × |
| Landon | 2005 | USA | cohort | 14529 | 10690 | × | × | × |  |  | × | × |  | × | × | × |  | × | × | × |
| Bujold | 2005 | USA | cohort | 6718 | 4786 | × | × |  |  | × |  |  |  |  |  |  |  | × |  | × |
| Thomas | 1994 | UAE | cohort | 704 | 554 |  |  |  |  |  |  |  |  | × |  |  |  |  |  | × |
| Coleman | 2001 | USA | cohort | 428 | 310 |  |  |  | × |  |  |  |  |  |  |  |  |  |  |  |
| Ilesanmi | 1997 | Nigeria | cohort | 113 | 98 |  |  |  |  |  |  |  |  |  |  | × |  | × |  |  |
| Caughey | 1998 | USA | cohort | 800 | 500 |  |  |  |  |  |  |  |  | × |  |  |  | × |  | × |
| Preveen | 1997 | Pakistan | cohort | 112 | 72 |  |  |  |  |  |  |  |  | × |  |  |  | × |  |  |
| McNally | 1999 | Ireland | cohort | 663 | 569 |  |  |  |  |  | × |  |  |  |  |  |  |  |  | × |
| Rageth | 1999 | Switzerland | cohort | 17613 | 12986 |  |  |  |  |  |  |  |  |  |  |  |  | × |  | × |
| Wasti | 1994 | Pakistan | cohort | 419 | 314 |  |  |  |  |  | × |  |  | × |  |  |  |  |  | × |
| Yasumizu | 1994 | Japan | cohort | 28 | 21 | × |  |  |  |  |  |  |  |  |  | × |  | × |  |  |
| Lovell | 1996 | UK | cross sectional | 244 | 197 | × |  |  |  |  |  |  |  |  |  | × |  | × |  |  |
| Obara | 1998 | Japan | cross sectional | 214 | 132 | × | × |  |  |  |  |  |  | × |  | × |  |  |  |  |
| Puliyath | 2009 | Oman | cohort | 370 | 277 | × |  |  |  |  | × |  | × |  |  |  |  | × |  |  |
| Holt | 1997 | USA | cohort | 6491 | 4007 |  |  |  | × | × |  |  |  | × |  |  |  | × |  | × |
| Srinivas | 2006 | USA | cohort | 13706 | 10350 |  |  |  |  | × |  |  |  |  |  |  |  |  |  |  |
| Huang | 2002 | USA | cohort | 1185 | 1007 |  |  |  |  |  |  |  | × |  |  |  |  |  |  |  |
