# Supplementary material for: Factors associated with successful vaginal birth after a cesarean section: a systematic review and meta-analysis
Source: BMC Pregnancy Childbirth. 2019 Oct 17;19:360. doi: 10.1186/s12884-019-2517-y (PMC6798397; doi:10.1186/s12884-019-2517-y)
Supplement: Supplementary file 20 — Additional file 20: Table S3. Newcastle-Ottawa quality assessment (DOCX 62 kb) [file 12884_2019_2517_MOESM20_ESM.docx]

| Supplementary Table S3. Study-specific Newcastle-Ottawa quality assessment | | | | | | | | | | |  |  |  |  |  |
| --- | --- | --- | --- | --- | --- | --- | --- | --- | --- | --- | --- | --- | --- | --- | --- |
| Table S3-1 Case-control studies | | | | | |  |  |  |  |  |  |  |  |  |  |
| Case-control studies | | |  | | Selection |  |  |  | Comparability |  |  | Exposure |  |  |  |
|  | |  | S1 | | S2 | S3 | S4 |  | C1 |  | E1 | E2 | E3 |  | Total |
|  | |  | Case | | Case | Control | Control |  | Confounder |  | Ascertainment | Method | Non- |  |  |
| Author | Year | | | validation | representativeness | selection | validation |  | adjustment |  |  | case/control | response |  |  |
| Birara | | 2013 | 1 | | 1 | 1 | 1 |  | 1 |  | 1 | 1 | 1 |  | 8 |
| Herman | | 2017 | 1 | | 1 | 1 | 0 |  | 1 |  | 1 | 1 | 1 |  | 7 |
| Table S3-1 Cohort studies | | | | | | |  |  |  |  |  |  |  |  |  |
| Cohort studies | | |  | | Selection |  |  |  | Comparability |  |  | Outcome |  |  |  |
|  | |  | S1 | | S2 | S3 | S4 |  | C1 |  | O1 | O2 | O3 |  | Total |
|  | |  | Exposed | | Non-exposed | Exposure | Outcome |  |  |  | Outcome | Follow | Losses |  |  |
| Author | | Year | representativeness | | Selection | ascertainment | baseline |  |  |  | assessment | up | follow-up |  |  |
| Miller | | 2015 | 1 | | 1 | 1 | 0 |  | 2 |  | 1 | 1 | 1 |  | 8 |
| Mirteymouri | | 2016 | 1 | | 1 | 1 | 0 |  | 0 |  | 1 | 1 | 1 |  | 6 |
| Kalisa | | 2017 | 1 | | 1 | 1 | 0 |  | 0 |  | 1 | 1 | 1 |  | 6 |
| Melamed | | 2013 | 1 | | 1 | 1 | 0 |  | 2 |  | 1 | 1 | 1 |  | 8 |
| Mizrachi | | 2017 | 1 | | 1 | 1 | 0 |  | 0 |  | 1 | 1 | 1 |  | 6 |
| Metz | | 2013 | 1 | | 1 | 1 | 0 |  | 0 |  | 1 | 1 | 1 |  | 6 |
| Tsai | | 2017 | 1 | | 1 | 1 | 0 |  | 0 |  | 1 | 1 | 1 |  | 6 |
| Siddiqui | | 2013 | 1 | | 1 | 1 | 0 |  | 1 |  | 1 | 1 | 1 |  | 7 |
| Black | | 2016 | 1 | | 1 | 1 | 1 |  | 0 |  | 1 | 1 | 1 |  | 7 |
| Balachandran | | 2014 | 1 | | 1 | 1 | 0 |  | 0 |  | 1 | 1 | 1 |  | 6 |
| Annessi | | 2016 | 1 | | 1 | 1 | 0 |  | 2 |  | 1 | 1 | 1 |  | 8 |
| Cameron | | 2004 | 1 | | 1 | 1 | 0 |  | 2 |  | 1 | 1 | 1 |  | 8 |
| Ashwal | | 2015 | 1 | | 1 | 1 | 0 |  | 2 |  | 1 | 1 | 1 |  | 8 |
| Beloosesky | | 2018 | 1 | | 1 | 1 | 1 |  | 0 |  | 1 | 1 | 1 |  | 7 |
| Bhide | | 2016 | 1 | | 1 | 1 | 0 |  | 2 |  | 1 | 1 | 1 |  | 8 |
| Smith | | 2005 | 1 | | 1 | 1 | 0 |  | 2 |  | 1 | 1 | 1 |  | 8 |
| Naji | | 2013 | 1 | | 1 | 1 | 1 |  | 0 |  | 1 | 1 | 1 |  | 7 |
| McDonald | | 2017 | 1 | | 1 | 1 | 0 |  | 2 |  | 1 | 1 | 1 |  | 8 |
| Fox | | 2018 | 1 | | 1 | 1 | 0 |  | 1 |  | 1 | 1 | 1 |  | 7 |
| Torralba | | 2017 | 1 | | 1 | 1 | 0 |  | 1 |  | 1 | 1 | 1 |  | 7 |
| Gonsalves | | 2016 | 1 | | 1 | 1 | 0 |  | 0 |  | 1 | 1 | 1 |  | 6 |
| Nkwabong | | 2016 | 1 | | 1 | 1 | 0 |  | 0 |  | 1 | 1 | 1 |  | 6 |
| Maykin | | 2017 | 1 | | 1 | 1 | 0 |  | 0 |  | 1 | 1 | 1 |  | 6 |
| Wen | | 2018 | 1 | | 1 | 1 | 0 |  | 0 |  | 1 | 1 | 1 |  | 6 |
| Yokoi | | 2012 | 1 | | 1 | 1 | 1 |  | 0 |  | 1 | 1 | 1 |  | 7 |
| Krispin | | 2018 | 1 | | 1 | 1 | 0 |  | 1 |  | 1 | 1 | 1 |  | 7 |
| Khan | | 2016 | 1 | | 1 | 1 | 0 |  | 0 |  | 1 | 1 | 1 |  | 6 |
| Senturk | | 2015 | 1 | | 1 | 1 | 0 |  | 2 |  | 1 | 1 | 1 |  | 8 |
| Singh | | 2015 | 1 | | 1 | 1 | 1 |  | 0 |  | 1 | 1 | 1 |  | 7 |
| Smriti | | 2014 | 1 | | 1 | 1 | 1 |  | 0 |  | 1 | 1 | 1 |  | 7 |
| Facchinetti | | 2015 | 1 | | 1 | 1 | 0 |  | 2 |  | 1 | 1 | 1 |  | 8 |
| Erez | | 2012 | 1 | | 1 | 1 | 0 |  | 0 |  | 1 | 1 | 1 |  | 6 |
| Knight | | 2013 | 1 | | 1 | 1 | 0 |  | 2 |  | 1 | 1 | 1 |  | 8 |
| Ouzounian | | 2011 | 1 | | 1 | 1 | 0 |  | 0 |  | 1 | 1 | 1 |  | 6 |
| Gonen | | 2004 | 1 | | 1 | 1 | 0 |  | 0 |  | 1 | 1 | 1 |  | 6 |
| Kwon | | 2009 | 1 | | 1 | 1 | 0 |  | 0 |  | 1 | 1 | 1 |  | 6 |
| Hollard | | 2006 | 1 | | 1 | 1 | 0 |  | 1 |  | 1 | 1 | 1 |  | 7 |
| Wanyonyi | | 2010 | 1 | | 1 | 1 | 0 |  | 0 |  | 1 | 1 | 1 |  | 6 |
| Ugwu | | 2014 | 1 | | 1 | 1 | 1 |  | 0 |  | 1 | 1 | 1 |  | 7 |
| Schoorel | | 2013 | 1 | | 1 | 1 | 0 |  | 0 |  | 1 | 1 | 1 |  | 6 |
| Tasleem | | 2015 | 1 | | 1 | 1 | 1 |  | 0 |  | 1 | 1 | 1 |  | 7 |
| Regan | | 2015 | 1 | | 1 | 1 | 0 |  | 2 |  | 1 | 1 | 1 |  | 8 |
| Paterson | | 1991 | 1 | | 1 | 1 | 0 |  | 0 |  | 1 | 1 | 1 |  | 6 |
| Comas | | 2016 | 1 | | 1 | 1 | 1 |  | 2 |  | 1 | 1 | 1 |  | 9 |
| Patel | | 2017 | 1 | | 1 | 1 | 0 |  | 0 |  | 1 | 1 | 1 |  | 6 |
| Nakamura | | 2017 | 1 | | 1 | 1 | 0 |  | 1 |  | 1 | 1 | 1 |  | 7 |
| Kalok | | 2017 | 1 | | 1 | 1 | 1 |  | 0 |  | 1 | 1 | 1 |  | 7 |
| Soni | | 2015 | 1 | | 1 | 1 | 1 |  | 0 |  | 1 | 1 | 1 |  | 7 |
| Shaheen | | 2014 | 1 | | 1 | 1 | 0 |  | 0 |  | 1 | 1 | 1 |  | 6 |
| Damle | | 2014 | 1 | | 1 | 1 | 0 |  | 0 |  | 1 | 1 | 1 |  | 6 |
| Sananes | | 2014 | 1 | | 1 | 1 | 1 |  | 1 |  | 1 | 1 | 1 |  | 8 |
| OBORO | | 2010 | 1 | | 1 | 1 | 0 |  | 0 |  | 1 | 1 | 1 |  | 6 |
| Olagbuji | | 2010 | 1 | | 1 | 1 | 1 |  | 0 |  | 1 | 1 | 1 |  | 7 |
| Weinstein | | 1996 | 1 | | 1 | 1 | 0 |  | 2 |  | 1 | 1 | 1 |  | 8 |
| Pathadey | | 2005 | 1 | | 1 | 1 | 0 |  | 0 |  | 1 | 1 | 1 |  | 6 |
| Jerbi | | 2006 | 1 | | 1 | 1 | 1 |  | 1 |  | 1 | 1 | 1 |  | 8 |
| Sujana | | 2017 | 1 | | 1 | 1 | 1 |  | 0 |  | 1 | 1 | 1 |  | 7 |
| Silva | | 2017 | 1 | | 1 | 1 | 0 |  | 2 |  | 1 | 1 | 1 |  | 8 |
| Faiz | | 2017 | 1 | | 1 | 1 | 0 |  | 0 |  | 1 | 1 | 1 |  | 6 |
| Alani | | 2017 | 1 | | 1 | 1 | 0 |  | 0 |  | 1 | 1 | 1 |  | 6 |
| Haumonte | | 2017 | 1 | | 1 | 1 | 0 |  | 0 |  | 1 | 1 | 1 |  | 6 |
| Puri | | 2011 | 1 | | 1 | 1 | 0 |  | 0 |  | 1 | 1 | 1 |  | 6 |
| Tripathi | | 2004 | 1 | | 1 | 1 | 1 |  | 0 |  | 1 | 1 | 1 |  | 7 |
| Dadhwal | | 2003 | 1 | | 1 | 1 | 0 |  | 0 |  | 1 | 1 | 1 |  | 6 |
| Obeidat | | 2013 | 1 | | 1 | 1 | 0 |  | 1 |  | 1 | 1 | 1 |  | 7 |
| Wong | | 2003 | 1 | | 1 | 1 | 1 |  | 0 |  | 1 | 1 | 1 |  | 7 |
| Madaan | | 2011 | 1 | | 1 | 1 | 1 |  | 0 |  | 1 | 1 | 1 |  | 7 |
| Khanum | | 2011 | 1 | | 1 | 1 | 1 |  | 0 |  | 1 | 1 | 1 |  | 7 |
| Marchiano | | 2004 | 1 | | 1 | 1 | 0 |  | 0 |  | 1 | 1 | 1 |  | 6 |
| Olusanya | | 2009 | 1 | | 1 | 1 | 0 |  | 0 |  | 1 | 1 | 1 |  | 6 |
| Bujold | | 2004 | 1 | | 1 | 1 | 0 |  | 1 |  | 1 | 1 | 1 |  | 7 |
| Gyamfi | | 2004 | 1 | | 1 | 1 | 0 |  | 1 |  | 1 | 1 | 1 |  | 7 |
| Grinstead | | 2004 | 1 | | 1 | 1 | 1 |  | 1 |  | 1 | 1 | 1 |  | 8 |
| Dinsmoor | | 2004 | 1 | | 1 | 1 | 0 |  | 0 |  | 1 | 1 | 1 |  | 6 |
| Durnwald | | 2004 | 1 | | 1 | 1 | 0 |  | 1 |  | 1 | 1 | 1 |  | 7 |
| Landon | | 2005 | 1 | | 1 | 1 | 1 |  | 1 |  | 1 | 1 | 1 |  | 8 |
| Bujold | | 2005 | 1 | | 1 | 1 | 0 |  | 2 |  | 1 | 1 | 1 |  | 8 |
| Thomas | | 1994 | 1 | | 1 | 1 | 0 |  | 0 |  | 1 | 1 | 1 |  | 6 |
| Coleman | | 2001 | 1 | | 1 | 1 | 0 |  | 0 |  | 1 | 1 | 1 |  | 6 |
| Ilesanmi | | 1997 | 1 | | 1 | 1 | 1 |  | 0 |  | 1 | 1 | 1 |  | 7 |
| Caughey | | 1998 | 1 | | 1 | 1 | 0 |  | 0 |  | 1 | 1 | 1 |  | 6 |
| Preveen | | 1997 | 1 | | 1 | 1 | 1 |  | 0 |  | 1 | 1 | 1 |  | 7 |
| McNally | | 1999 | 1 | | 1 | 1 | 1 |  | 0 |  | 1 | 1 | 1 |  | 7 |
| Rageth | | 1999 | 1 | | 1 | 1 | 1 |  | 0 |  | 1 | 1 | 1 |  | 7 |
| Wasti | | 1994 | 1 | | 1 | 1 | 0 |  | 0 |  | 1 | 1 | 1 |  | 6 |
| Yasumizu | | 1994 | 1 | | 1 | 1 | 1 |  | 0 |  | 1 | 1 | 1 |  | 7 |
| Lovell | | 1996 | 1 | | 1 | 1 | 0 |  | 0 |  | 1 | 1 | 1 |  | 6 |
| Obara | | 1998 | 1 | | 1 | 1 | 0 |  | 0 |  | 1 | 1 | 1 |  | 6 |
| Puliyath | | 2009 | 1 | | 1 | 1 | 1 |  | 0 |  | 1 | 1 | 1 |  | 7 |
| Holt | | 1997 | 1 | | 1 | 1 | 1 |  | 0 |  | 1 | 1 | 1 |  | 7 |
| Srinivas | | 2006 | 1 | | 1 | 1 | 0 |  | 2 |  | 1 | 1 | 1 |  | 8 |
| Huang | | 2002 | 1 | | 1 | 1 | 1 |  | 0 |  | 1 | 1 | 1 |  | 7 |
