# Supplementary material for: Factors associated with successful vaginal birth after a cesarean section: a systematic review and meta-analysis
Source: BMC Pregnancy Childbirth. 2019 Oct 17;19:360. doi: 10.1186/s12884-019-2517-y (PMC6798397; doi:10.1186/s12884-019-2517-y)
Supplement: Supplementary file 21 — Additional file 21: Table S4.Subgroup analysis of factors (DOCX 73 kb) [file 12884_2019_2517_MOESM21_ESM.docx]

Table S4 Subgroup analysis of factors

| Factor | Type | Subgroup 1 | Subgroup 2 | Studies, N | SMD/OR (95%) | I^2^ (%) | *P*-value |
| --- | --- | --- | --- | --- | --- | --- | --- |
| Age | SMD | Size | <100 | 7 | 0.04 (-0.48, 0.55) | 80.5 | <0.001 |
|  |  |  | 100-1000 | 22 | -0.06 (-0.14, 0.02) | 46.1 | 0.010 |
|  |  |  | >1000 | 7 | -0.04 (-0.16, 0.09) | 96.3 | <0.001 |
|  |  | Location | Asia | 20 | -0.01 (-0.13, 0.11) | 60.5 | <0.001 |
|  |  |  | Africa | 3 | 0.18 (0.06, 0.30) | 0.0 | 0.606 |
|  |  |  | Europe | 7 | -0.21 (-0.29, -0.13) | 73.4 | 0.001 |
|  |  |  | North America | 6 | 0.02 (-0.02, 0.05) | 62.6 | <0.626 |
|  |  | Design | Cohort | 31 | -0.05 (-0.12, 1.03) | 80.8 | <0.001 |
|  |  |  | Cross sectional | 5 | -0.21 (-0.23, -0.18) | 0.0 | 0.421 |
| BMI | SMD | Date | Pre-pregnancy | 3 | -0.36 (-0.50, -0.22) | 0 | 0.777 |
|  |  |  | At first prenatal visit | 2 | -0.17 (-0.27, -0.07) | 0 | 0.595 |
|  |  |  | At admission before delivery | 11 | -0.34 (-0.42, -0.25) | 26.6 | 0.191 |
|  | OR | Date | Continous Variable | 4 | 0.95 (0.92, 0.98) | 0 | 0.749 |
|  |  |  | Obesity | 10 | 0.50 (0.39, 0.64) | 84.9 | <0.001 |
|  |  |  | Other comparison | 3 | 0.39 (0.16, 0.98) | 74.9 | 0.019 |
| Obesity | OR | Size | <500 | 4 | 0.46 (0.26, 0.83) | 0 | 0.405 |
|  |  |  | 500-1000 | 3 | 0.45 (0.13, 1.55) | 96.1 | <0.001 |
|  |  |  | >1000 | 3 | 0.57 (0.53, 0.60) | 1.3 | 0.363 |
|  |  | Location | North America | 4 | 0.59 (0.52, 0.69) | 65.1 | 0.035 |
|  |  |  | Asia | 5 | 0.26 (0.11, 0.64) | 72.7 | 0.005 |
|  |  |  | Oceania | 1 | 0.72 (0.46, 1.12) | - | - |
|  |  | Design | All are cohort studies. |  |  |  |  |
| BMI | Adjusted OR | Date | Continous Variable | 4 | 0.95 (0.93, 0.98) | 73.1 | 0.011 |
|  |  |  | Binary Variable | 2 | 0.52 (0.37, 0.72) | 0 | 0.349 |
|  |  | Size | <2000 | 3 | 0.96 (0.91, 1.00) | 79.1 | 0.008 |
|  |  |  | >2000 | 1 | 0.95 (0.93, 0.96) | - | - |
|  |  | Location | North America | 2 | 0.93 (0.90, 0.97) | 64.4 | 0.094 |
|  |  |  | Europe | 2 | 0.98 (0.95, 1.00) | 47.9 | 0.166 |
|  |  | Design | All are cohort studies. |  |  |  |  |
| Smoke | OR | Size | <5000 | 4 | 1.11 (0.72, 1.73) | 71.8 | 0.014 |
|  |  |  | >5000 | 2 | 1.16 (1.09, 1.23) | 0 | 0.871 |
|  |  | Location | Europe | 2 | 1.55 (0.75, 3.19) | 75.1 | 0.045 |
|  |  |  | North America | 3 | 1.01 (0.77, 1.32) | 74.1 | 0.021 |
|  |  |  | Asia | 1 | 0.97 (0.44, 2.12) | - | - |
|  |  | Design | All are cohort studies. |  |  |  |  |
| Diabetes | OR | Size | <1000 | 9 | 0.49 (0.33, 0.73) | 35.1 | 0.137 |
|  |  |  | 1000-5000 | 6 | 0.53 (0.43, 0.65) | 0 | 0.526 |
|  |  |  | 5000-10000 | 4 | 0.54 (0.38, 0.77) | 81.1 | 0.001 |
|  |  |  | >10000 | 2 | 0.36 (0.30, 0.44) | 0 | 0.482 |
|  |  | Location | Europe | 5 | 0.40 (0.35, 0.46) | 0 | 0.510 |
|  |  |  | Asia | 4 | 0.59 (0.27, 1.25) | 1.7 | 0.384 |
|  |  |  | North America | 12 | 0.56 (0.46, 0.69) | 50.4 | 0.023 |
|  |  | Design | Only one is case control study. Others are cohort. |  |  |  |  |
| HDCP | OR | Size | <500 | 7 | 0.26 (0.13, 0.54) | 9.0 | 0.360 |
|  |  |  | 500-5000 | 3 | 0.63 (0.49, 0.81) | 0 | 0.426 |
|  |  |  | 5000-8000 | 2 | 0.49 (0.20, 1.23) | 98.2 | <0.001 |
|  |  |  | >8000 | 2 | 0.68 (0.56, 0.81) | 30.5 | 0.230 |
|  |  | Location | Asia | 3 | 0.10 (0.03, 0.32) | 0 | 0.989 |
|  |  |  | North America | 6 | 0.72 (0.66, 0.79) | 4.0 | 0.391 |
|  |  |  | Europe | 4 | 0.40 (0.24, 0.66) | 68.6 | 0.023 |
|  |  |  | Africa | 1 | 1.30 (0.16, 10.57) | - | - |
|  |  | Design | Only one study is cross sectional. Others are cohort. |  |  |  |  |
| Previous VB(before CS) | OR | Size | <500 | 24 | 3.28 (2.50, 4.30) | 44.9 | 0.010 |
|  |  |  | 500-5000 | 12 | 2.72 (1.84, 4.04) | 88.3 | <0.001 |
|  |  |  | >5000 | 3 | 4.51 (4.09, 4.98) | 61.3 | 0.075 |
|  |  | Location | Africa | 4 | 2.91 (0.74, 11.36) | 91.7 | <0.001 |
|  |  |  | Asia | 19 | 3.22 (2.32, 4.47) | 68.0 | <0.001 |
|  |  |  | Europe | 8 | 3.15 (1.73, 5.74) | 91.5 | <0.001 |
|  |  |  | North America | 8 | 3.44 (2.76, 4.28) | 82.5 | <0.001 |
|  |  | Design | Only one study is case control. Others are cohort. | - | - | - | - |
|  | Adjusted OR | Size | <1000 | 11 | 3.13 (2.00, 4.91) | 71.4 | <0.001 |
|  |  |  | 1000-10000 | 4 | 2.61 (2.06, 3.32) | 0 | 0.582 |
|  |  |  | >10000 | 1 | 4.20 (3.87, 4.56) | - | - |
|  |  | Location | Europe | 5 | 3.00 (1.92, 4.70) | 44.4 | 0.126 |
|  |  |  | Asia | 5 | 2.63 (1.31, 5.27) | 81.4 | <0.001 |
|  |  |  | North America | 5 | 3.18 (2.23, 4.53) | 62.2 | 0.032 |
|  |  |  | Africa | 1 | 5.49 (2.23, 13.49) | - | - |
|  |  | Design | All are cohort studies. |  |  |  |  |
| Inter-delivery interval | SMD | Size | <100 | 4 | -0.35 (-1.00, 0.29) | 32.7 | 0.216 |
|  |  |  | 100-500 | 6 | -0.08 (-0.33, 0.17) | 72.2 | 0.003 |
|  |  |  | >500 | 2 | -0.38 (-0.74, -0.02) | 89.5 | 0.002 |
|  |  | Location | Asia | 9 | 0.03 (-0.26, 0.32) | 88.5 | <0.001 |
|  |  |  | North America | 1 | -0.19 (-0.37, -0.09) | - | - |
|  |  |  | Africa | 1 | 0.46 (-0.30, 1.23) | - | - |
|  |  |  | Europe | 1 | 0.32 (0.11, 0.54) | - | - |
|  |  | Design | cohort | 10 | -0.01 (-0.26, 0.24) | 88.5 | <0.001 |
|  |  |  | Cross sectional | 2 | 0.38 (0.08, 0.68) | 0 | 0.771 |
|  | OR | Size | <1000 | 8 | 1.39 (0.95, 2.03) | 67.0 | 0.003 |
|  |  |  | >1000 | 3 | 1.17 (0.83, 1.64) | 97.2 | <0.001 |
|  |  | Location | Asia | 6 | 1.55 (0.93, 2.59) | 72.1 | 0.003 |
|  |  |  | Europe | 2 | 1.13 (0.59, 2.14) | 60.2 | 0.113 |
|  |  |  | Africa | 1 | 0.88 (0.93, 2.59) | - | - |
|  |  |  | North America | 2 | 1.34 (1.23, 1.45) | - | - |
|  |  | Design | Cohort | 9 | 1.25 (0.98, 1.60) | 91.1 | <0.001 |
|  |  |  | Cross sectional | 1 | 2.63 (1.08, 6.40) | - | - |
|  |  |  | Case control | 1 | 0.88 (0.61, 1.26) | - | -- |
|  | Adjusted OR |  | Only 3 studies. |  |  |  |  |
| White race | OR | Size | <1000 | 7 | 1.36 (1.11, 1.67) | 14.3 | 0.321 |
|  |  |  | 1000-10000 | 5 | 1.46 (1.10, 1.94) | 86.4 | <0.001 |
|  |  |  | >10000 | 2 | 1.40 (1.25, 1.55) | 84.4 | 0.011 |
|  |  | Location | North America | 8 | 1.42 (1.17, 1.72) | 79.5 | <0.001 |
|  |  |  | Europe | 5 | 1.43 (1.19, 1.72) | 51.4 | 0.084 |
|  |  |  | Oceania | 1 | 1.15 (0.76, 1.73) | - | - |
|  |  | Design | All are cohort studies. |  |  |  |  |
| Black race | OR | Size | <1000 | 6 | 0.58 (0.42, 0.81) | 58.5 | 0.034 |
|  |  |  | 1000-10000 | 3 | 0.82 (0.71, 0.94) | 0 | 0.464 |
|  |  |  | >10000 | 2 | 0.19 (0.02, 2.34) | 100 | <0.001 |
|  |  | Location | North America | 7 | 0.67 (0.55, 0.82) | 68.6 | 0.004 |
|  |  |  | Europe | 4 | 0.35 (0.06, 2.03) | 99.2 | <0.001 |
|  |  | Design | All are cohort studies. |  |  |  |  |
| Asian race | OR | Size | <1000 | 2 | 0.73 (0.24, 2.20) | 79.7 | 0.026 |
|  |  |  | 1000-10000 | 2 | 0.59 (0.45, 0.77) | 0 | 0.356 |
|  |  |  | >10000 | 1 | 0.81 (0.77, 0.85) | - | - |
|  |  | Location | North America | 3 | 0.60 (0.28, 1.29) | 65.8 | 0.054 |
|  |  |  | Europe | 2 | 0.73 (0.56, 0.95) | 74.0 | 0.050 |
|  |  | Design | All are cohort studies. |  |  |  |  |
| Latina race | OR | Size | <1000 | 4 | 0.64 (0.44, 0.95) | 62.5 | 0.046 |
|  |  |  | 1000-5000 | 2 | 0.87 (0.58, 1.29) | 56.0 | 0.132 |
|  |  |  | >5000 | 1 | 0.65 (0.59, 0.72) | - | - |
|  |  | Location | All studies are from North America. |  |  |  |  |
|  |  | Design | All are cohort studies. |  |  |  |  |
| HDCP* | OR | Size | <300 | 3 | 1.00 (0.25, 3.91) | 50.2 | 0.135 |
|  |  |  | >300 | 2 | 0.54 (0.15, 1.98) | 89.8 | 0.002 |
|  |  | Location | All studies are from Asia. |  |  |  |  |
|  |  | Design | All studies are cohort. |  |  |  |  |
| Dystocia/ Failure to progress* | OR | Size | <500 | 21 | 0.51 (0.42, 0.62) | 25.0 | 0.144 |
|  |  |  | 500-1000 | 8 | 0.68 (0.34, 1.38) | 95.9 | <0.001 |
|  |  |  | >1000 | 2 | 0.27 (0.09, 0.84) | 98.5 | <0.001 |
|  |  | Location | Asia | 19 | 0.55 (0.38, 0.79) | 81.1 | <0.001 |
|  |  |  | North America | 6 | 0.75 (0.41, 1.39) | 96.2 | <0.001 |
|  |  |  | Europe | 4 | 0.35 (0.16, 0.76) | 90.8 | <0.001 |
|  |  |  | Africa | 2 | 0.25 (0.06, 1.12) | 52.3 | 0.147 |
|  |  | Design | cohort | 28 | 0.52 (0.39, 0.69) | 91.3 | <0.001 |
|  |  |  | Cross sectional | 3 | 0.77 (0.52, 1.14) | 0 | 0.845 |
| Fetal distress* | OR | Size | <500 | 19 | 1.06 (0.83, 1.36) | 59.3 | 0.001 |
|  |  |  | 500-1000 | 7 | 0.90 (0.64, 1.27) | 70.7 | 0.002 |
|  |  |  | >1000 | 2 | 1.03 (0.84, 1.27) | 81.6 | 0.020 |
|  |  | Location | Asia | 19 | 0.94 (0.77, 1.15) | 45.0 | 0.018 |
|  |  |  | North America | 6 | 1.00 (0.82, 1.21) | 70.5 | 0.005 |
|  |  |  | Africa | 3 | 1.74 (0.57, 5.30) | 85.8 | 0.001 |
|  |  | Design | Cohort | 26 | 1.03 (0.88, 1.20) | 63.8 | <0.001 |
|  |  |  | Cross sectional | 1 | 0.95 (0.53, 1.71) | - | - |
|  |  |  | Case control | 1 | 0.59 (0.29, 1.18) | - | - |
| Fetal malpresentation* | OR | Design | <500 | 19 | 1.71 (1.34, 2.19) | 43.2 | 0.024 |
|  |  |  | 500-1000 | 6 | 1.47 (0.90, 2.40) | 84.9 | <0.001 |
|  |  |  | >1000 | 2 | 2.14 (1.98, 2.32) | 0 | 0.600 |
|  |  | Location | Asia | 18 | 1.60 (1.28, 1.99) | 38.7 | 0.048 |
|  |  |  | Africa | 3 | 1.59 (0.48, 5.30) | 81.0 | 0.005 |
|  |  |  | North America | 6 | 1.76 (1.26, 2.47) | 91.4 | <0.001 |
|  |  | Design | Cohort | 23 | 1.74 (1.42, 2.14) | 77.6 | <0.001 |
|  |  |  | Cross sectional | 3 | 1.48 (0.97, 2.27) | 0 | 0.449 |
|  |  |  | Case control | 1 | 0.89 (0.44, 1.84) | - | - |
| Dystocia/ Failure to progress* | OR | Size | <500 | 5 | 0.45 (0.32, 0.64) | 5.2 | 0.377 |
|  |  |  | >500 | 3 | 0.42 (0.13, 1.43) | 95.7 | <0.001 |
|  |  | Location | Europe | 3 | 0.21 (0.13, 0.33) | 41.5 | 0.181 |
|  |  |  | North America | 3 | 0.64 (0.44, 0.93) | 30.2 | 0.239 |
|  |  |  | Africa | 1 | 0.69 (0.28, 1.69) | - | - |
|  |  |  | Asia | 1 | 0.80 (0.31, 2.07) | - | - |
|  |  | Design | All are cohort studies. |  |  |  |  |
| Birth weight | SMD | Size | <100 | 8 | -0.28 (-0.55, -0.01) | 45.7 | 0.075 |
|  |  |  | 100-500 | 14 | -0.26 (-0.46, -0.06) | 83.1 | <0.001 |
|  |  |  | 500-1000 | 3 | -0.25 (-0.40, -0.11) | 51.0 | 0.130 |
|  |  |  | >1000 | 7 | -0.21 (-0.28, -0.14) | 84.5 | <0.001 |
|  |  | Location | Asia | 14 | -0.21 (-0.34, -0.07) | 53.4 | 0.009 |
|  |  |  | Africa | 5 | -0.23 (-0.59, 0.12) | 80.1 | <0.001 |
|  |  |  | Europe | 6 | -0.33 (-0.53, -0.14) | 91.4 | <0.001 |
|  |  |  | North America | 7 | -0.22 (-0.31, -0.13) | 64.1 | 0.010 |
|  |  | Design | All are cohort. |  |  |  |  |
| Macrosomia | OR | Size | <500 | 9 | 0.25 (0.12,0.51) | 52.2 | 0.033 |
|  |  |  | 500-5000 | 6 | 0.56 (042,0.75) | 68.8 | 0.007 |
|  |  |  | >5000 | 6 | 0.59 (0.51,0.68) | 92.7 | <0.001 |
|  |  | Location | Asia | 6 | 0.33 (0.18, 0.60) | 48.5 | 0.084 |
|  |  |  | Africa | 4 | 0.19 (0.05, 0.74) | 60.6 | 0.055 |
|  |  |  | North America | 7 | 0.60 (0.50, 0.72) | 83.4 | <0.001 |
|  |  |  | Europe | 3 | 0.57 (0.44, 0.73) | 91.2 | <0.001 |
|  |  |  | Oceania | 1 | 0.74 (0.64, 0.84) | - | - |
|  |  | Design | Only 1 study is cross sectional. Others are cohort. |  |  |  |  |
| Gestational week | SMD | Size | <100 | 5 | 0.18 (-0.08, 0.44) | 0 | 0.601 |
|  |  |  | 100-1000 | 17 | -0.14 (-0.22, -0.06) | 36.9 | 0.063 |
|  |  |  | 1000-5000 | 5 | -0.09 (-0.37, 0.18) | 95.8 | <0.001 |
|  |  |  | >5000 | 2 | -0.07 (-0.42, 0.29) | 99.6 | <0.001 |
|  |  | Location | North America | 7 | -0.14 (-0.25, -0.04) | 78.1 | <0.001 |
|  |  |  | Asia | 15 | -0.09 (-0.20, 0.02) | 39.6 | 0.057 |
|  |  |  | Europe | 5 | 0.06 (-0.07, 0.20) | 75.2 | 0.003 |
|  |  |  | Africa | 2 | -0.05 (-1.21, 1.11) | 88.8 | 0.003 |
|  |  | Design | cohort | 26 | -0.07 (-0.17, 0.04) | 93.3 | <0.001 |
|  |  |  | Cross sectional | 3 | -0.32 (-0.51, -0.13) | 0 | 0.658 |
|  | OR | comparison | Continuous variable | 1 | 0.97 (0.84, 1.12) | - | - |
|  |  |  | GW≥37 vs. GW<37 | 4 | 0.99 (0.82, 1.19) | 0 | 0.396 |
|  |  |  | GW≥40 vs. GW<40 | 6 | 0.72 (0.40, 1.31) | 84.5 | <0.001 |
|  |  |  | GW≥41 vs. GW<41 | 3 | 0.65 (0.40, 1.07) | 69.5 | 0.038 |
| GW≥40 vs. GW<40 | OR | Size | <500 | 4 | 0.52 (0.20, 1.37) | 69.0 | 0.022 |
|  |  |  | >500 | 2 | 1.01 (0.46, 2.21) | 92.8 | <0.001 |
|  |  | Location | Asia | 2 | 0.22 (0.09, 0.53) | 0 | 0.378 |
|  |  |  | Africa | 2 | 1.07 (0.61, 1.88) | 0 | 0.751 |
|  |  |  | Oceania | 2 | 1.01 (0.46, 2.21) | 92.8 | <0.001 |
|  |  | Design | Cohort | 4 | 0.80 (0.38, 1.66) | 86.6 | <0.001 |
|  |  |  | Cross sectional | 1 | 0.28 (0.10, 0.80) | - | - |
|  |  |  | Case control | 1 | 1.02 (0.54, 1.93) | - | - |
| Induction of labor | OR | Size | <1000 | 25 | 0.54 (0.40, 0.73) | 80.7 | <0.001 |
|  |  |  | 1000-10000 | 12 | 0.60 (0.47, 0.76) | 93.8 | <0.001 |
|  |  |  | >10000 | 3 | 0.63 (0.49, 0.82) | 95.8 | <0.001 |
|  |  | Location | Asia | 16 | 0.53 (0.35, 0.81) | 85.9 | <0.001 |
|  |  |  | North America | 14 | 0.64 (0.52, 0.79) | 92.8 | <0.001 |
|  |  |  | Europe | 8 | 0.56 (0.46, 0.69) | 78.9 | <0.001 |
|  |  |  | Oceania | 1 | 0.90 (0.59, 1.37) | - | - |
|  |  |  | Africa | 1 | 0.34 (0.24, 0.48) | - | - |
|  |  | design | Cohort | 38 | 0.62 (0.54, 0.70) | 86.1 | <0.001 |
|  |  |  | Cross sectional | 2 | 0.22 (0.09, 0.50) | 88.0 | 0.004 |
| Induction of labor | Adjusted OR | Size | <1000 | 4 | 0.61 (0.38, 0.97) | 67.6 | 0.026 |
|  |  |  | >1000 | 5 | 0.55 (0.42, 0.71) | 79.0 | 0.001 |
|  |  | Location | North America | 5 | 0.58 (0.49, 0.70) | 56.5 | 0.056 |
|  |  |  | Europe | 2 | 0.37 (0.20, 0.67) | 58.3 | 0.121 |
|  |  |  | Oceania | 1 | 1.14 (0.67, 1.94) | - | - |
|  |  |  | Asia | 1 | 0.62 (0.40, 0.97) | - | - |
|  |  | Design | All studies are cohort. |  |  |  |  |
| Epidural anesthesia | OR | Size | <1000 | 5 | 0.52 (0.16, 1.66) | 93.4 | <0.001 |
|  |  |  | >1000 | 3 | 1.18 (0.41, 3.36) | 98.9 | <0.001 |
|  |  | Location | Asia | 4 | 0.75 (0.31, 1.83) | 86.2 | <0.001 |
|  |  |  | North America | 3 | 1.24 (0.43, 3.59) | 98.9 | <0.001 |
|  |  |  | Oceania | 1 | 0.11 (0.06, 0.18) | - | - |
|  |  | Design | All studies are cohort. |  |  |  |  |

*Indications for previous cesarean section.

SMD: standardized mean difference; OR: Odds Ratio; BMI: body mass index; HDCP: hypertensive disorders complicating pregnancy; VB: vaginal birth; VBAC: vaginal birth after cesarean; CS: cesarean section; CPD: cephalopelvic disproportion; BW: birth weight; GW: gestational week.
