# Supplementary material for: Factors associated with successful vaginal birth after a cesarean section: a systematic review and meta-analysis
Source: BMC Pregnancy Childbirth. 2019 Oct 17;19:360. doi: 10.1186/s12884-019-2517-y (PMC6798397; doi:10.1186/s12884-019-2517-y)
Supplement: Supplementary file 24 — Additional file 24: Appendix 1. Search strategy. (DOCX 26 kb) [file 12884_2019_2517_MOESM24_ESM.docx]

Appendix S1 Search strategy

| Database | Search strategy | Number of papers | total | After removing duplicate records |
| --- | --- | --- | --- | --- |
| MEDLINE (Ovid) | exp Vaginal Birth after Cesarean/ OR tolac.ab,ti OR vbac.ab,ti OR ((vaginal birth* OR vaginal deliver* OR trial of labor OR trial of labour OR active labor OR active labour) adj4 (cesarean* OR caesarean* OR postcesarean OR postcaesarean OR c section* OR abdominal deliver* OR uterine scar*)).ab,ti | 4,402 | 10,042 | 5,745 |
| Embase (Elsevier)  (1974 -) | ('vaginal birth after cesarean'/de OR tolac:ab,ti OR vbac:ab,ti OR (('vaginal birth' OR 'vaginal births' OR 'vaginal delivery' OR 'vaginal deliveries' OR 'trial of labor' OR 'trial of labour' OR 'active labor' OR 'active labour') NEAR/4 (cesarean* OR caesarean* OR postcesarean OR postcaesarean OR 'c section' OR 'c sections' OR 'abdominal delivery' OR 'abdominal deliveries' OR 'uterine scar' OR 'uterine scars')):ab,ti)  NOT 'conference abstract'/it | 3,392 |  |  |
| CINAHL Plus (EBSCO) | (MH "Vaginal Birth After Cesarean") OR TI (tolac OR vbac OR (("vaginal birth*" OR "vaginal deliver*" OR "trial of labor" OR "trial of labour" OR "active labor" OR "active labour") N4 (cesarean* OR caesarean* OR postcesarean OR postcaesarean OR "c section*" OR "abdominal deliver*" OR "uterine scar*"))) OR AB (tolac OR vbac OR (("vaginal birth*" OR "vaginal deliver*" OR "trial of labor" OR "trial of labour" OR "active labor" OR "active labour") N4 (cesarean* OR caesarean* OR postcesarean OR postcaesarean OR "c section*" OR "abdominal deliver*" OR "uterine scar*"))) | 2,248 |  |  |
